# X-ray Diffraction Evidence for Low Force Actin-Attached and Rigor-Like Cross-Bridges in the Contractile Cycle

**DOI:** 10.3390/biology5040041

**Published:** 2016-10-26

**Authors:** Felicity Eakins, Christian Pinali, Anthony Gleeson, Carlo Knupp, John M. Squire

**Affiliations:** 1 Faculty of Medicine, Imperial College London, Exhibition Road, London SW7 2AZ, UK; felicity.eakins@gmail.com (F.E.); KnuppC@Cardiff.ac.uk (C.K.); 2 Biophysics Group, Optometry & Vision Sciences, University of Cardiff, Cardiff CF10 3XQ, UK; christian.pinali@manchester.ac.uk; 3 Daresbury Laboratory, Warrington WA4 4AD, UK; anthony.gleeson@stfc.ac.uk; 4 Muscle Contraction Group, School of Physiology, Pharmacology & Neuroscience, Faculty of Biomedical Sciences, University of Bristol, Bristol BS8 1TH, UK

**Keywords:** tetanus rising phase, equatorial X-ray diffraction, sarcomere length control, myosin cross-bridge cycle, crossbridge time-course modelling

## Abstract

Defining the structural changes involved in the myosin cross-bridge cycle on actin in active muscle by X-ray diffraction will involve recording of the whole two dimensional (2D) X-ray diffraction pattern from active muscle in a time-resolved manner. Bony fish muscle is the most highly ordered vertebrate striated muscle to study. With partial sarcomere length (SL) control we show that changes in the fish muscle equatorial A-band (10) and (11) reflections, along with (10)/(11) intensity ratio and the tension, are much more rapid than without such control. Times to 50% change with SL control were 19.5 (±2.0) ms, 17.0 (±1.1) ms, 13.9 (±0.4) ms and 22.5 (±0.8) ms, respectively, compared to 25.0 (±3.4) ms, 20.5 (±2.6) ms, 15.4 (±0.6) ms and 33.8 (±0.6) ms without control. The (11) intensity and the (10)/(11) intensity ratio both still change ahead of tension, supporting the likelihood of the presence of a head population close to or on actin, but producing little or no force, in the early stages of the contractile cycle. Higher order equatorials (e.g., (30), (31), and (32)), more sensitive to crossbridge conformation and distribution, also change very rapidly and overshoot their tension plateau values by a factor of around two, well before the tension plateau has been reached, once again indicating an early low-force cross-bridge state in the contractile cycle. Modelling of these intensity changes suggests the presence of probably two different actin-attached myosin head structural states (mainly low-force attached and rigor-like). No more than two main attached structural states are necessary and sufficient to explain the observations. We find that 48% of the heads are off actin giving a resting diffraction pattern, 20% of heads are in the weak binding conformation and 32% of the heads are in the strong (rigor-like) state. The strong states account for 96% of the tension at the tetanus plateau.

## 1. Introduction

Despite tremendous progress in understanding the contractile mechanism in muscle and other myosin motors (Wulf et al. [1]; Squire et al. [2]), it is still uncertain exactly how many myosin heads interact at the same time with actin filaments in muscle to utilise the energy associated with ATP hydrolysis and produce muscular contraction. It is also not known how many different attached states there are and how much force is produced by each state. What is known is that in all muscles myosin (M) with ATP bound and hydrolysed to ADP and inorganic phosphate (Pi) in the form M.ADP.Pi can attach to actin filaments after the muscles have been switched on and that, while attached, the products Pi and then ADP are released [3]. During some stage of this process force and movement (if the muscle is free to shorten) are produced. Binding of another ATP molecule splits the heads from actin and a further cycle of hydrolysis and actin attachment can then take place.

The myosin molecule has a long rod-shaped region, a two chain coiled-coil α-helix, on the end of which are two elongated globular heads which carry the ATP hydrolysis and actin binding sites of the molecule. The myosin rods form the shaft or backbone of myosin filaments and the myosin heads project from the surface of this backbone and can bind to and interact with adjacent actin filaments (see overview in [2]). This much we know. However, what are the structural changes in the myosin heads that are associated with force production and movement? The myosin head has two major domains within it, a globular *motor domain* which is the enzymatic and actin attachment part of the head, and a long *lever arm* consisting of a long α–helix surrounded by two light chains. Crystal structures show that the motor and lever arm are linked through a small region called the *converter domain* [4,5,6,7] and this has revealed two main kinds of structural difference between states. One is the marked difference in the angle at which the lever arm comes out of the motor domain. The other is the opening and closing of a cleft in the motor domain [7,8,9]. It is now thought that movement is brought about by the swinging of the lever arm around a relatively fixed myosin motor domain bound to actin [4,5]. However, at what stage is force produced? What is the shape of the myosin head when it first attaches to actin? Does the lever arm of attached heads swing through a series of steps or are there, say, only two main structural attached states of the head, together with elasticity in some part of the head (e.g., in the lever arm or perhaps converter/transducer domains)?

Low-angle X-ray diffraction patterns from a variety of muscles have been recorded with ever increasing success over a number of decades ([10,11,12,13,14,15,16,17,18,19,20,21,22] and many others; see [23,24,25] for overviews and many additional references). Much of the improvement has been due to the exploitation of ever more powerful synchrotron radiation sources and the development of very fast 2D X-ray detectors [26]. Important and unambiguous information has come from these studies. However, in no case have the diffraction data from the whole low-angle 2D diffraction pattern been modelled fully at every stage of the contractile cycle.

The disadvantage of the technique of X-ray diffraction is that it does not provide direct images of the diffracting object, the diffraction patterns need to be interpreted and modelled. These diffraction patterns carry a wealth of information, but they can sometimes be over-interpreted. For example, in a series of papers, the so-called M3 reflection of the meridian of diffraction patterns from frog muscles (the M3 corresponds to the 14.3 nm repeat of “crowns” of myosin heads in the myosin filaments), was used in an attempt to deduce the angles that the lever arms of myosin heads make on the motor domain under different conditions [27,28,29,30,31,32,33,34,35,36,37,38,39,40]; for further references see [41]. Unfortunately, this was not unambiguous, and we showed [41] that these results were simply reporting the relative axial positions of different motor domains in different populations of myosin heads without specifying the location of the lever arm.

Interpretation of diffraction data is not trivial and a very helpful pre-requisite is that the muscle doing the diffracting is very highly ordered so that it gives well-sampled X-ray diffraction patterns. Muscles of choice are the indirect flight muscles of insects such as the giant water bug *Lethocerus* [42,43], and the muscles of bony fish which for the vertebrates are the most highly regular muscles [44,45]. Bony fish muscle order is such that all the myosin filaments in a given A-band have exactly the same rotation around their long axes, so that their crowns of heads make a quasi-crystalline three-dimensional (3D) array. This is a simple lattice muscle [46]. Other vertebrate muscles such as those in frogs, rabbits, chickens, humans and all higher vertebrates have a so-called statistical superlattice A-band where the myosin filaments can have one of two rotations around their long axes, but these rotations are not regularly distributed across the A-band [45,46,47,48,49]. Simple lattice muscles give highly sampled myosin diffraction patterns [15,44,50], but superlattice muscles do not, and their X-ray patterns are, therefore, intrinsically more difficult to solve.

In previous work on bony fish muscles using either static muscles [44] or in a time-resolved way [15,24,44,51,52], we suggested (as have others using other muscles) that there is a population of heads, possibly weak-binding heads, which attach transiently to actin at an early stage in the contractile cycle, but which do not yet produce high levels of force. These changes were recorded without SL control and any muscle shortening during the contraction would complicate the interpretation of what is happening. The present paper outlines our approach to determining the full 2D low-angle X-ray diffraction pattern from Plaice fin muscle in a time-resolved way so that the contractile mechanism can be fully modelled. We sought to optimise as much as possible about this preparation, including the dissection step, the Ringer solution used and the nature of the electrical stimulus. We also tested the effects of sarcomere length control. In this paper, we present the methods and results from this optimization. The whole 2D diffraction pattern was recorded out to about *d* = 60 Å. Here we describe the resulting diffraction data from the equator of the diffraction pattern and we model the results using a new procedure. Results from other parts of the diffraction pattern will be presented elsewhere.

## 2. Material and Methods

### 2.1. Specimen Preparation and Optimisation

#### 2.1.1. Plaice Fin Muscle Anatomy and Dissection

Preparations from the teleost *Pleuronectes platessa* (Plaice) were single intact fin muscles whose role is to aid control of the dorsal and anal fins of the flatfish [49,53] and to help the fish to bury themselves in the seabed and to streamline their shape against strong currents. Several muscles are associated with each bone in the fin (Supplementary Materials Figure S1). Fin muscles just under the skin were always used because the skin provided a convenient attachment point for one end of the muscle and there was a single tendon connection to bone at the other end. Flatfish are particularly good for this type of preparation because the dorsal and anal fins cover the whole length of their bodies and comprise a side-by-side array of many similar muscles. Several of the required muscles can be obtained from a single fish. In this study the fin muscles were typically 15 to 20 mm long and up to 3 mm in diameter.

Flatfish from the London University Marine Biological Station (U.M.B.S.), Isle of Cumbrae, Scotland or from Aquarium Technologies Ltd. (Weymouth, UK), were kept alive in tanks of re-circulating sea water at 5–7 °C for up to one week. Details of the anatomy of the fin muscles and their dissection are given here in Supplementary Material (Figures S2–S5; [54]). Briefly, the fish were stunned by a blow to the head, and killed by destruction of the brain. Several small blocks of the dorsal and anal fin tissue and bones, approximately 5 or 6 muscles long, were removed from each fish and placed in aerated Ringer solution on ice. Two viable muscle preparations were usually obtained from each block of tissue, and several muscles from one fish. Specimens were moistened and chilled by pipetting fresh iced Ringer solution onto them at regular intervals during the dissection. A probe was used to loosen the gap between fin muscles and the skin, the skin was removed and the muscle was cleared of fat. A small slit was made in the skin at the top of the muscle for the tension transducer hook to pass through. Muscles were pinned out onto individual pieces of rubber (see Supplementary Materials Figure S5), and immersed in a beaker of fresh aerated Ringer solution, kept on ice; they could be used without any obvious deterioration of performance for up to 15 h after dissection.

#### 2.1.2. Optimising the Contractile Response

In the past we have used a modified Cobb Ringer with or without calcium [55], as in Supplementary Materials Table S1 and reported in Harford and Squire [15,44]. However, other Ringers have been used for other fish muscles such as Sculpin [55,56,57,58]. We tested four different Ringers with the same electrical stimulus protocol to see which would give the largest peak tetanic tension and the greatest resistance to fatigue. These were the modified Cobb Ringer with or without calcium, the James Ringer and the Hudson Ringer. Unfortunately, the original Cobb Ringer [55] had problems with calcium salt precipitation and pH variation. During dissection and storage of muscles, but not during the X-ray exposures, the Ringer solution was oxygenated. Because of a fast activation response and relatively slow fatigue rate achieved from initial tests, the Ringer from James et al. [57] was the preferred Ringer and was used throughout. Supplementary Materials Figure S6 shows that Ringer composition has a major effect not only on the maximum tension achieved but also on the rise time of tetanic contractions and the speed of relaxation.

Optimal settings were found for electrical stimulus, including voltage (17 V), frequency (140 Hz) and shape of the stimulating pulses (pulse width 0.08 ms). Optimal rest length was found to be at 2.3 µm which correlates well with the optimum SL obtained by James and Johnston [56] in sculpin.

### 2.2. Design of a Length Control System for Whole Plaice Fin Muscle

#### 2.2.1. Mounting Muscle Preparations

The dissected muscles were mounted in Perspex X-ray chambers as shown in Supplementary Materials Figure S7. A slot at the top of the chamber allowed the lever arm of a transducer (Aurora Scientific Inc. (Aurora, ON, Canada) 300B Lever Arm tension and length transducer) to enter the chamber and connect to the skin end of the muscle preparation. A syringe allowed air to be bubbled through the Ringer solution. After mounting the muscle the whole chamber was filled with 10 mL of fresh cold Ringer solution. A copper cooling jacket, cooled by a water mixture from a Neslab RTE-7 refrigerated bath, kept the temperature between 7 °C and 8 °C for all experiments.

#### 2.2.2. SL Control System

It is well known [57,58] that during end-held isometric contractions in striated muscle changes in sarcomere length (SL) can occur. These changes can slow the apparent development of tension as series connective tissues lengthen and sarcomeres shorten and take up slack within the muscle. Changes in SL can also affect the intensities of reflections in the muscle X-ray diffraction pattern [59], in particular on the equator. In the case of bony fish muscle, the SL change accompanying end-held contractions had not been measured and it was not known whether any SL changes which do occur affect the intensity time-courses of the muscle X-ray reflections. Harford and Squire [15] argued that the SL changes should be small in bony fish muscle (at most 4%), based on measurements of A-band mass during contraction which changed very little. Supplementary Materials Figure S8 shows the control system we designed to have: a spatial resolution of 0.002 µm or better; the ability to reduce SL change to less than 3% of the original SL; a response time to SL change of a millisecond or less. Of the several methods available [14,60,61,62,63] we chose to use a laser diffractometer. To allow the laser and X-ray beams to be collinear, a very thin mylar mirror with an aluminium flash was designed (see Supplementary Materials Figure S9). Position sensitive detectors (see Supplementary Materials Figure S10) were used to track the positions of the two first order peaks, giving highly accurate measurements of their movements.

#### 2.2.3. Use of Two Reflections versus One

Most laser diffraction sarcomere control systems only record the position of one of the first order diffraction peaks (e.g., [64,65]). These studies assume that both left and right first order peaks move equally during changes in SL and that the zero order peak position remains fixed in position. There are different views on the safety of this [60,61,62,63,65,66,67,68]. In the present experiments, the movement of the zero order laser reflection was measured during typical isometric contractions (Supplementary Materials Figure S11), showing that a movement of around 0.5 mm can occur in the zero order reflection at a specimen to detector distance of 160.5 mm. This movement in the whole diffraction pattern would cause an erroneous reading of 0.024 µm in the SL measurement if only one order was measured; ten times larger than the required accuracy of the instrument as stated above. This problem was eliminated by measuring the positions of the two first order reflections with the light source at normal incidence. Additionally, both the laser beam and X-ray beam must shine through the same part of the specimen; a detector to measure the zero order laser peak would block the X-ray beam. If the laser beam was angled to just intersect the X-ray beam within the specimen, as in some studies [69], the X-ray and laser beams would follow different paths and sample different muscle volumes. Here the use of a reflecting surface in the X-ray beam enabled the laser and X-ray beams to sample exactly the same region of the muscle.

At the beginning of each muscle experiment the SL of the muscle was accurately set; a detachable mirror was positioned in the gap between the X-ray cell and detector housing to reflect the laser pattern upwards onto a more distant screen (14.29 cm; Supplementary Materials Figure S12) where the SL could be set accurately.

The two first order laser diffraction lines were then collected onto position-sensitive detectors positioned so that their output was zeroed at the set resting SL. When required, fluctuations from this length were reduced by a negative feedback system (see Supplementary Materials Figure S8).

### 2.3. Recording X-ray Diffraction Data

All time-resolved X-ray diffraction data were recorded on beam-line 16.1 at the then CCLRC Daresbury Synchrotron Radiation Source (SRS) using the RAPID X-ray detector [70,71]. Station 16.1 provided high intensity X-rays at a fixed wavelength of 1.41 Å and beam size of typically 1000 µm × 500 µm. The RAPID detector on line 16.1 was a multi-wire proportional counter (MWPC) detector employing “wire MicroGap” technology to give a much higher count rate performance than conventional MWPCs ([70]; global count rate greater than 2 × 10^7^ photons per second; maximum local count rate 10^6^ photons·mm^−2^·s^−1^; two orders of magnitude greater than conventional MWPC detectors at that time; active area 200 mm × 200 mm; spatial resolution 200 µm). A fast shutter was employed just before the specimen to keep the exposure of the muscle to X-rays to a minimum and an ionisation chamber also positioned before the specimen was used to monitor the X-ray flux reaching the muscle.

**Figure 1:**
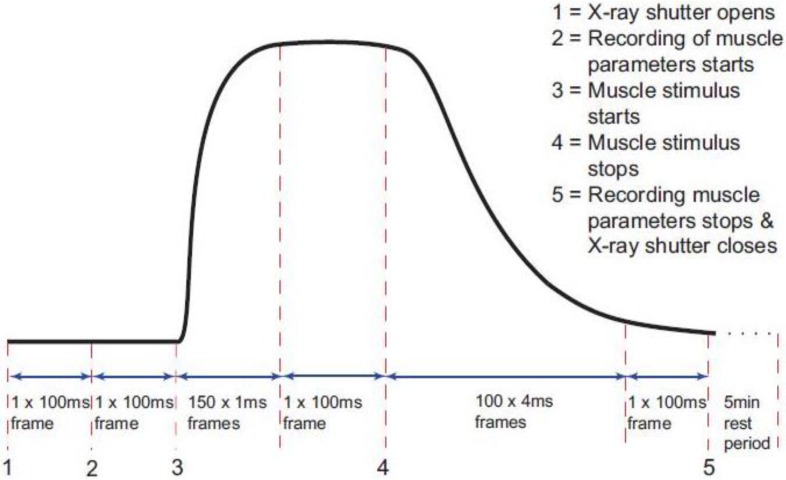
Diagram of the timing protocol used for time-resolved X-ray diffraction experiments. Black trace: a diagrammatic representation of the tetanic tension record produced by an isometrically contracting muscle showing the exposure times for the X-ray diffraction patterns taken throughout the contraction (red dashed lines). The numbers 1 to 5 indicate points where an action occurs as in the top right hand corner of the figure. X-ray data and ionisation chamber readings were recorded throughout. The other muscle parameters such as the muscle tension, change in length and sarcomere length (SL) were only recorded between 2 and 5.

The timing protocol in Figure 1 was used to record time-resolved X-ray data during isometric contractions under the control of the beam-line acquisition system. Frame exposures were: 2 × 10 ms, 150 × 1 ms, 1 × 100 ms, 100 × 4 ms, 1 × 100 ms and 1 × 300,000 ms (Figure 1). The rising phase of the contraction occurred during the 150 × 1 ms frames and in the present paper this is the part of the contraction which is being investigated. Up to 90 contractions were performed on each muscle, with cumulative data from each 10 contractions summed and saved as a single time series file of 254 successive X-ray diffraction patterns or frames with exposure times given in Figure 1. Fifteen muscles were used with SL control giving 780 contractions and 12 muscles without control giving 620 contractions. The X-ray data were collected in two different experimental sessions, and in each case data were recorded with and without SL control. Sixty-second blank chamber (with Ringer and no muscle) camera patterns recorded regularly throughout the beam-time were scaled and removed from the muscle diffraction patterns during data processing. As well as recording the diffraction data, the beam-line acquisition system saved the total ionisation chamber reading for each X-ray frame during the contraction. The muscle tension, change in SL and stimulus were all recorded via a data acquisition card in a PC running an acquisition program designed in the test and measurement software Testpoint (version 3.4; CEC Capital Equipment, Bedford, NH, USA) which was itself controlled by the beam-line acquisition system so that all parts of the experiment and data logging were synchronised.

### 2.4. Analysis of the X-Ray Data

Analysis of the time-resolved X-ray data was carried out using the programs FibreFix version 1.3 [72,73] and Peakfit version 4 from AISN Software Inc. (Florence, OR, USA). The counts recorded by the X-ray detector for each pixel in the pattern are proportional to the intensity of the X-rays incident on that pixel.

#### 2.4.1. Centring and Alignment

To allow X-ray time series from different muscles to be added together the patterns were centred and rotationally aligned using FibreFix by first estimating the centre, rotation and tilt of the pattern by selecting the positions of particular reflections. Values were refined using the Refine tool and all the frames in each time series were centred and rotated using the “ROT” tool to make the equator and meridian respectively exactly horizontal and vertical. In some of the time series the rotation of the X-ray diffraction pattern changed slightly but suddenly during contractions. The “PLA” tool allowed each frame in the series to be displayed in sequence to identify the frames at which the rotation change occurred. The “ROT” tool was then used to orient the frames as necessary. Time series recorded during the two different experimental sessions at the beam-line were not added at this stage due to possible differences in camera length. Four summed time series were obtained from this step in the processing, two from each experimental session for the two datasets with and without SL control. Negative pixels occurring in the patterns after subtraction of the blank camera pattern were zeroed using the “ZER” tool. The negatives were always very small and only arose in the short exposure frames where some pixels held zero values in the raw pattern. Before background fitting was carried out, the “MIR” tool was used on the summed time series to average the contents of all four quadrants of a frame so that it becomes symmetric across the equator and meridian.

#### 2.4.2. Background Subtraction

Diffuse background scatter was removed from the patterns to leave only the Bragg reflections using the 2D background fitting algorithms in Fibrefix. Because of the weakness of the sampled myosin layer-lines, the 100 ms active frame was found to give the best background fit, with no detectable reflection intensity in the fitted background. This background was subtracted from all the other frames in the time series. The smooth background based on the method developed by Ivanova and Makowski [74] was applied to the active frame. A circularly symmetric background was then fitted three times over, using different fitting parameters each time. This method worked very well for the first experimental beam-time. However, for the second experimental session, the background, although of the same form, increased in intensity slightly as the muscle contracted, so the background was scaled throughout the time series to give a better fit to the diffraction patterns.

#### 2.4.3. Conversion to Reciprocal Space and Peak Fitting

Following background subtraction the frames were converted to reciprocal space using the “FTOREC” tool in Fibrefix. This put all the time series onto the same spatial scale and allowed the files from different experimental sessions to be added together using the “ADD” tool. This resulted in two final time series, one with and one without SL control. The program Peakfit was used to fit peaks to one-dimensional (1D) data. To obtain the integrated intensities of the equatorial reflections throughout the time series, 1D vertically integrated profiles were taken of the equator for each frame in the time series using the “VER” tool. This produced 2 × 254 1D equatorial profiles. Any remaining background in the 1D profiles was fitted through points between the peaks using the expression *y* = Ae^B*x*^ + C*x*^4^ + D*x*^3^ + E*x*^2^ + F*x* + G, where A, B, C, D, E, F and G are all constants, *x* represents the position in pixels and *y* the intensity in counts. Once a satisfactory 1D background fit had been obtained, Gaussian profiles for the nine peaks visible in the profile were added in. The spacing of the M3 reflection, carefully measured previously for relaxed bony fish muscle to be 143.2 Å [44], was used to convert pixel positions to reciprocal Angstroms.

### 2.5. Equatorial Time-Course Modelling

**Figure 2:**
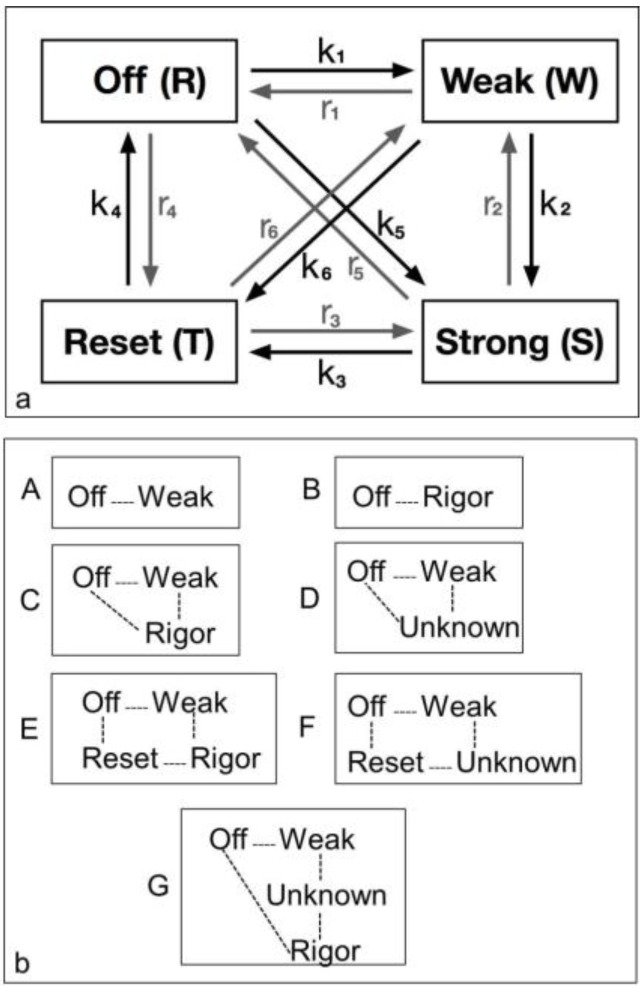
Possible models for the crossbridge cycle including 4 or fewer structural states and the transitions between them. For convenience the states have been given names. (**a**) A general model for the crossbridge cycle including four possible states and transitions between them. However, they can be considered in different ways as in (**b**); (**b**) Various kinds of model (A to G) have been considered as described in the text. These include two 2-state models (A, B), two 3-state models (C, D) and three 4-state models (E, F, G).

The diagram in Figure 2 shows all possible state transitions for the myosin heads. Initially all heads were in the resting state (or Off). Resting heads were allowed to change their state to a weak binding state (Weak) with rate constant *k*_1_ or to a resetting state (Reset) with rate constant *r*_4_ or directly to a strongly bound state (Strong) with rate constant *k*_5_ (see arrows). In turn, weakly bound heads could go back to a resting state (with rate constant *r*_1_), move to a strongly bound state (with rate constant *k*_2_) or to a resetting state (with rate constant *k*_6_). Analogously, heads in the strong and resetting state can change their state as indicated by the arrows in the figure with the appropriate rate constants. Mathematically, this can be expressed with a system of first order differential equations. Indicating with *R*(*t*), *W*(*t*), *S*(*t*) and *T*(*t*) the percentage of heads in the resting, weakly bound, strongly bound and resetting state, respectively, at time t, we have:

d*R*(*t*)/d*t* = −*k*_1_*R*(*t*) + *r*_1_*W*(*t*) − *r*_4_*R*(*t*) + *k*_4_*T*(*t*) − *k*_5_*R*(*t*) + *r*_5_*S*(*t*), (1)

d*W*(*t*)/d*t* = −*r*_1_*W*(*t*) + *k*_1_*S*(*t*) − *k*_2_*W*(*t*) + *r*_2_*S*(*t*) − *k*_6_*W*(*t*) + *r*_6_*T*(*t*), (2)

d*S*(*t*)/d*t* = −*r*_2_*S*(*t*) + *k*_2_*W*(*t*) − *k*_3_*S*(*t*) + *r*_3_*T*(*t*) − *r*_5_*S*(*t*) + *k*_5_*R*(*t*), (3)

d*T*(*t*)/d*t* = −*r*_3_*T*(*t*) + *k*_3_*S*(*t*) − *k*_4_*T*(*t*) + *r*_4_*R*(*t*) − *r*_6_*T*(*t*) + *k*_6_*W*(*t*), (4)

For each choice of the rate constants *k*_1_, ..., *k*_6_, *r*_1_, …, *r*_6_, the system was then solved numerically using the Runge-Kutta fourth order method with a time step of 1 ms and all heads being in the resting state at time *t* = 0. This gave the percentage of heads in each state, every millisecond, for 150 ms after muscle activation. The predicted X-ray intensities at time *t* (indicated below as *I*_hk_(*t*)) for the (10), (11), (20), (21), (30), (31), (32) and (40) reflections) were calculated from the amplitudes of each reflection as:

*I*_10_(*t*) = (*A*_10R_*R*(*t*) + *A*_10W_*W*(*t*) + *A*_10S_*S*(*t*) + *A*_10T_*T*(*t*))^2^, (5)

*I*_11_(*t*) = (*A*_11R_*R*(*t*) + *A*_11W_*W*(*t*) + *A*_11S_*S*(*t*) + *A*_11T_*T*(*t*))^2^, (6)

*I*_40_(*t*) = (*A*_40R_*R*(*t*) + *A*_40W_*W*(*t*) + *A*_40S_*S*(*t*) + *A*_40T_*T*(*t*))^2^, (7)

with *A*_10R_ the amplitude of the (10) reflection in the resting state, *A*_10W_ the amplitude of the (10) reflection in the weak binding state, etc. These amplitudes were found either experimentally (e.g., from diffraction patterns of resting or rigor muscles) see Supplementary Material Table S2, or, as in the case of the rate constants, by fitting the intensities of the time course X-ray data.

The best values for all unknown parameters were found by fitting the time course by a simulating annealing fitting process [75]. Using the notation *I*_10ex_(*t*) to indicate the intensity of the (10) reflection obtained experimentally at time *t*, and *I*_10_(*t*) the intensity calculated as described above (and using an analogous notation for all other reflections), the function to minimise during the simulated annealing process was:

Chi = ∑*_t_* {*w*_10_(*I*_10_(*t*) − *I*_10ex_(*t*))^2^ + *w*_11_(*I*_11_(*t*) − *I*_11ex_(*t*))^2^ + ... + *w*_40_(*I*_40_(*t*) − *I*_40ex_(*t*))^2^}, (8)

where the sum extended over the whole time course (*t* = 1, …, 150 ms). *w*_10_, ..., *w*_40_ are weight factors appropriately chosen to correct for the intrinsic noise and reduced reliability of the higher order reflections.

## 3. Results

### 3.1. Recording and Control of Sarcomere Length Changes

Isometric tetani (Figure 1) were induced in dissected single Plaice fin muscles (Supplementary Materials Figures S1–S5 and S7), and sarcomere length control was attempted using the method of laser diffraction and continuous feedback (Supplementary Materials Figures S5 and S7–S13). It is clear from the trace without feedback applied (Supplementary Materials Figure S13) that the laser diffraction system can follow changes in SL very well indeed. The time to 50% change in SL was about 10 to 20 ms, very similar to the tension response. The absolute change without SL control from 2.27 to 2.20 µm (average uncontrolled reduction in length 0.0727 ± 0.0012 µm, a change of 3.19% ± 0.06%) was close to the suggestion of Harford and Squire [15]. With continuous monitoring and feedback applied, the SL reduction was very much less: 0.0364 ± 0.0004 µm or 1.58% ± 0.02% giving 18.2 nm/half sarcomere. Results from Reconditi et al. [76] on single fibres of frog *tibialis anterior* muscle, where tetanus rising phase X-ray time-courses were also reported, involved a SL reduction of −20 nm/half sarcomere; our results and methods on whole muscle were at least as effective as theirs on single muscle fibres.

The final tensions achieved were 123.7 (*±*4.0) kN·m*^−^*^2^ (*n* = 38: all the X-ray experiments reported here). Individual tensions without and with SL control were 120.3 (*±*4.2) kN·m*^−^*^2^ (*n* = 14) and 125.5 (*±*5.8) kN·m*^−^*^2^ (*n* = 24), not significantly different (*p* = 0.54). Muscle fatigue reduced these values by about 10% in the first ten contractions.

### 3.2. Equatorial X-Ray Diffraction from Resting and Active Muscle

**Figure 3:**
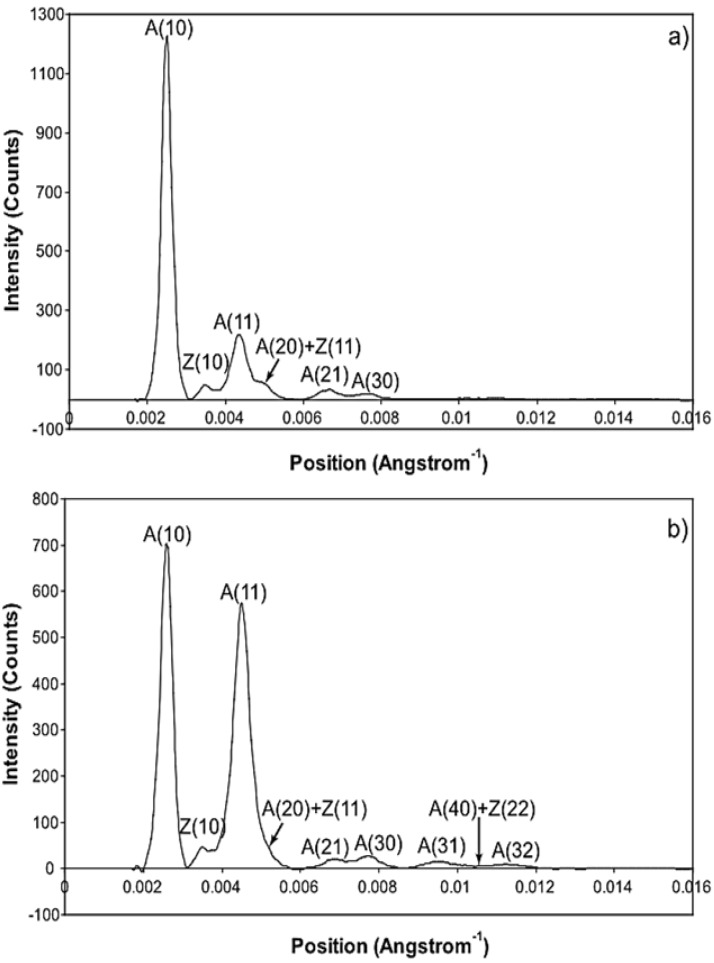
Equatorial profiles from the 100 ms relaxed and active frames of the summed time series with SL control. Both profiles were vertically integrated from 0.00125 Å^−1^ on either side of the equator. Data from 780 contractions were summed to produce the two profiles giving a total exposure time of 78 s. The background scatter was removed from the profiles to leave just the Bragg peaks by fitting a polynomial plus exponential function to it. (A: A-band peaks; Z: Z-band peaks). (**a**) Equatorial profile from the 100 ms relaxed frame. Six equatorial peaks are visible in the relaxed profile; (**b**) Equatorial profile from the 100 ms active frame. Nine equatorial peaks are visible in the active profile.

The whole of the low-angle X-ray diffraction pattern from Plaice fin muscle was recorded out to a spacing of about 60 Å throughout the timing protocol shown in Figure 1 and the data were processed as discussed in Materials and Methods using FibreFix and Peakfit. Here we consider the average time-resolved changes on the equator of the diffraction pattern during the rising phase and tension plateau of the tetanus. Figure 3 shows the observed intensity peaks on the equator in Plaice fin muscle X-ray diffraction patterns from the resting (Figure 3a) and tension plateau (Figure 3b) phases in the timing protocol of Figure 1. Reflections out to the A(32) peak are indicated. Note that peaks come from both the A-band and the Z-line in the muscle; they are identified here by A or Z before the reflection indices [77,78]. As reported in many previous studies, the main changes are the drop in intensity of the A(10) reflection and the increase in the A(11) peak from rest to active muscle (e.g., [15,18,21,59,69,79] and many others).

**Figure 4:**
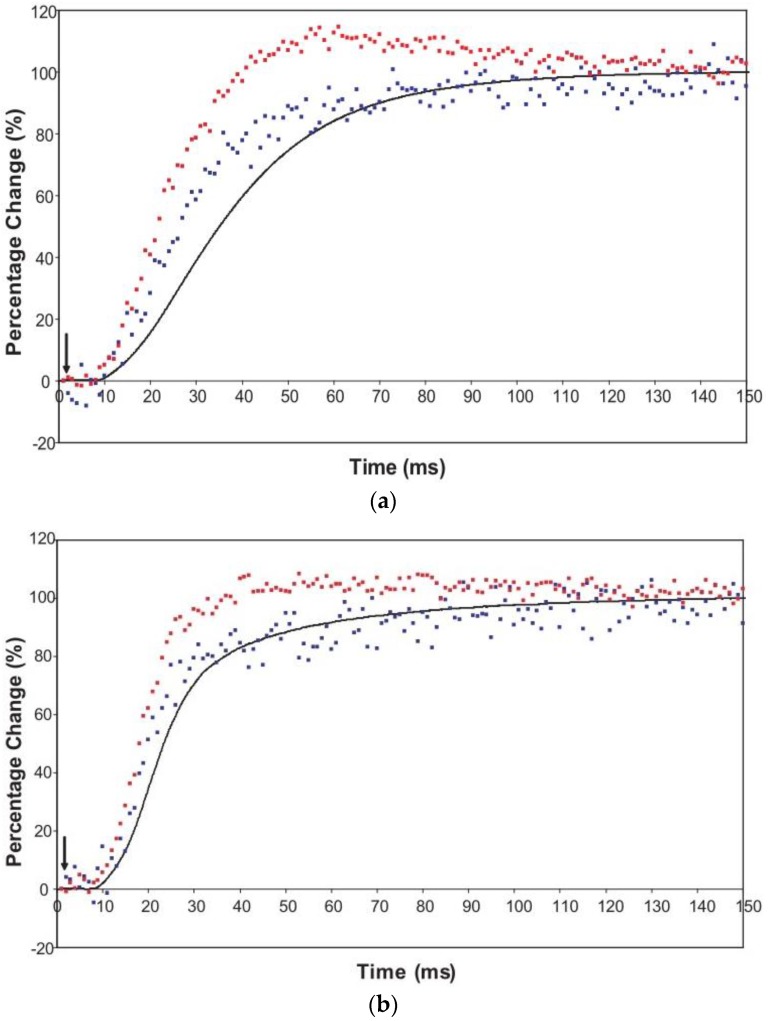
Average time-courses of the A(10) and A(11) intensities and tension during the 150 ms rising phase of isometric tetanic contraction in Plaice fin muscle without and without SL control. The inverted A(10) time-course, actually a drop in intensity, is shown in blue, the A(11) increase in red and the tension increase in black. Time-courses have been normalised with respect to their average values over 100 ms in the relaxed state (0%) and 100 ms at the plateau of contraction (100%) to show the percentage of maximum change. Activation occurs at the position of the black arrow. (**a**) Time-courses of the A(10) and A(11) intensities and tension without SL control; (**b**) Time-courses of the A(10) and A(11) intensities and tension with partial SL control.

The time courses of the changes in these intensities during the rising phase of a tetanus are shown in Figure 4 for muscle without (Figure 4a) or with (Figure 4b) SL control.

Here the A(10) intensity drop and the A(11) intensity increase have been shown as relative changes normalised to 100% at the tension plateau. This is so that their time-courses can be compared directly with that of the recorded tension which has also been normalised to the same scale. What is apparent in (a), as reported by Harford and Squire [15], is a marked lead of the A(11) and A(10) intensity changes over the tension change, with the A(11) change leading the A(10) change. As before, the A(11) time-course also showed a slight overshoot before settling down to the active plateau level. This was thought to provide evidence for attachment of myosin heads to actin on activation without them at first producing a great deal of tension, possibly in a non-force-producing, weak-binding state. The heads would then later switch over to a force producing state and tension would increase.

**Figure 5:**
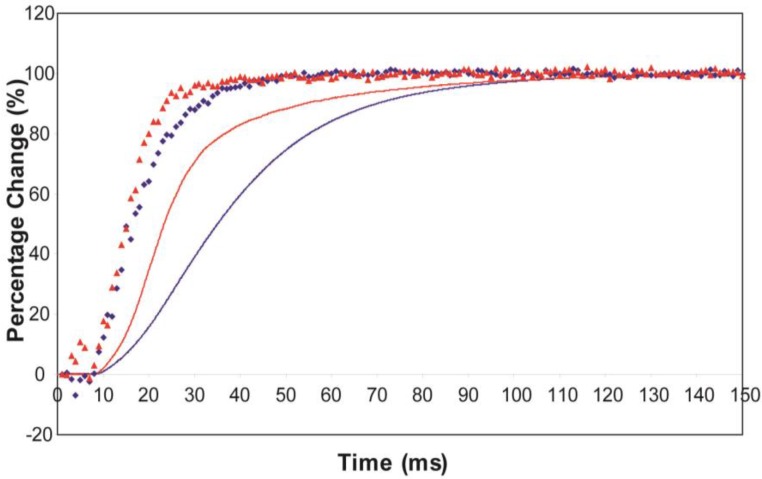
Plots of the A(10) to A(11) intensity ratio (intensity of (10)/intensity of (11), written as I(10)/I(11)) and tension during the rising phase of tetanic contraction in Plaice fin muscle for both datasets under the two different SL conditions (without control in blue, with partial SL control in red, tension solid lines, intensity ratio discrete points). For comparison the time-courses have been normalised (as in Figure 8) with respect to their average values over 100 ms in the relaxed state (0%) and 100 ms at the plateau of contraction (100%) to show the percentage of maximum change. There is a very clear lead of intensity changes ahead of tension.

The effect of using partial SL control, as shown in Figure 4b, is to make all the observed changes very much faster, but to retain the general sequence of events. The A(11) change still leads the A(10) change, which still leads tension, but everything is shifted to shorter times. In addition, the A(11) peak time-course still has a different profile to those of the A(10) and tension in that it reaches a high value very quickly, almost overshooting as in Figure 4a but not so dramatically, and then stays fairly constant while the A(10) intensity and the tension are still rising to their plateau values. Figure 5 shows the time-course of the change in the intensity ratio of the A(10) to the A(11) peaks compared to tension in experiments with (red) and without (blue) SL control. In both cases, the lead between the X-ray changes and the tension is very marked.

**Figure 6:**
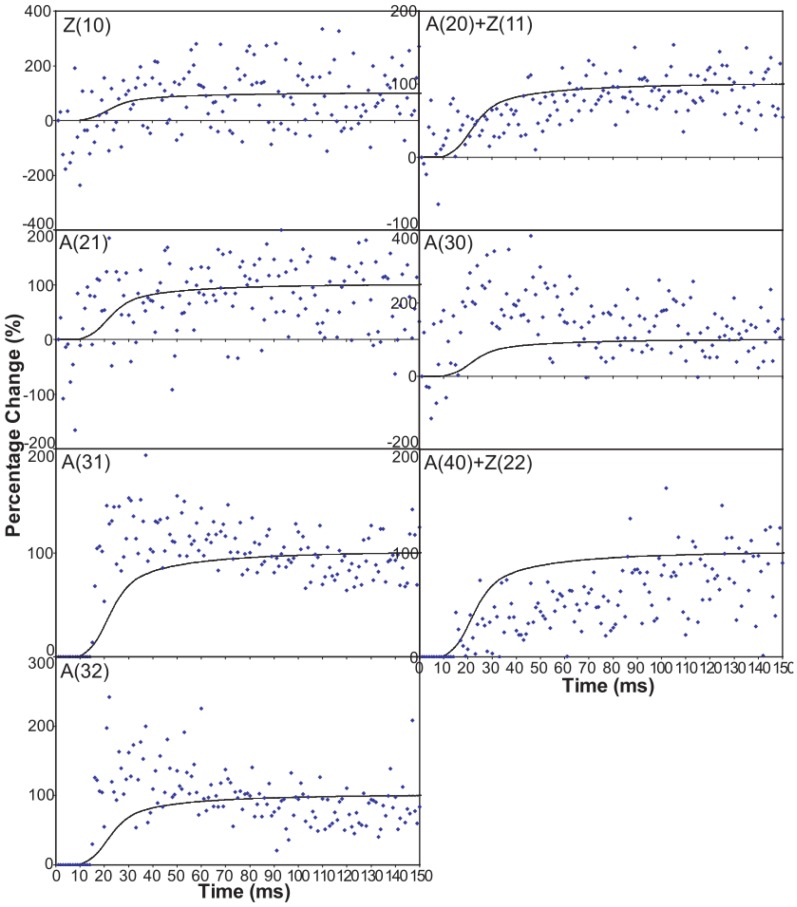
Intensity time-courses of the equatorial reflections, other than the A(10) and A(11), visible in the time series with partial SL control. The time-course of each reflection is shown on a separate plot in blue, as labelled, with the tension as a continuous line in grey. For comparison, the time-courses have been normalised with respect to their average values over 100 ms in the relaxed state (0%) and 100 ms at the plateau of contraction (100%) to show percentage of maximum change as in Figure 4 and Figure 5.

Figure 6 shows time-courses for the higher order equatorial peaks in Figure 3, including both the Z-line and A-band peaks, all normalised and shown relative to the tension time-course. These are all relatively weak reflections compared with the A(10) and A(11) peaks and the signal is therefore rather noisier. However, in some cases there are very clear changes. For example, the A(30), A(31) and A(32) peaks all change rapidly (increase) and show a clear overshoot before settling to their tension plateau values. There are Z-line contributions to the equator as detailed in Harford et al. [78], but these are dominated by the ordered lengths of actin filaments each side of the Z-band. The Z(10) and Z(11) may be fairly strong, but the reflections outside that are relatively weak, presumably mainly because of filament disorder. Even then, the Z(10) is on its own is observed to be relatively weak in Figure 3 and shows only small smooth changes in Figure 6. The Z(11) overlaps the A(20) peak and together they are relatively weak. The Z(20) overlaps the A(21) peak. This also shows small smooth changes without overshoots. The overshoots are only observed in the A(11), A(30), A(31) and A(32) peaks which do not involve the main Z-line peaks. In summary, from knowledge of the Z-band structure [78], it is likely that the Z-band peaks beyond the Z(10) and Z(11) will be relatively weak and there is no apparent reason why they should change a great deal in active muscle, so their possible minor contributions have been ignored in the present analysis.

## 4. Discussion

### 4.1. Plaice Fin Muscle Handling and Properties

The immediate aim of this work was to record the changing intensities of the equatorial peaks from bony fish muscle in a time-resolved way, improved by SL control and improved muscle treatment, and to model the results to learn about crossbridge behaviour. However, it was also to establish whole bony fish muscle as a viable preparation to study the whole of the low-angle X-ray diffraction patterns from myosin and actin filaments in active muscle. The muscle of choice for this project was that of bony fish skeletal muscle because of its simple lattice structure and well-sampled resting X-ray diffraction patterns [44]. The performance of the active fin muscle specimens was improved to increase their tension output and to reduce their rate of fatigue (see Materials and Methods Section 2.1 and the Supplementary Materials Section S2). A method was also developed to set up a SL measurement and control system for active whole Plaice fin muscles; muscles large enough to give reasonable diffraction statistics even with relatively weak reflections.

We wanted to assess the effect of SL change in active bony fish muscle because SL is known to have an effect on both the time-course of tension development and the changes in equatorial X-ray intensities in other muscles [59,69]. Previous work carried out on bony fish muscle neglected SL changes when interpreting the relationship between tension and X-ray intensity time-courses from tetanically contracting muscle [15,24,51,80]. Until this current project the contractile SL change in bony fish muscle had not been measured and the extent to which such changes might affect the tension and X-ray time-courses and the conclusions drawn from them about the cross-bridge cycle was not known definitively.

Table 1 compares the time responses of various muscles during the rising phase of tetanic contractions with the new results in the present paper. Immediately apparent is that Plaice fin muscles under SL control at 7 to 8 °C show a very fast response; the time to half tension change is 22.5 ± 0.8 ms compared to 30 to 50 ms in other intact, electrically stimulated, muscles.

### 4.2. Deductions about the Cross-Bridge Mechanism

Here, we evaluate what the observed equatorial intensity changes mean in terms of changes in myosin cross-bridge organisation during the contractile cycle. We are concerned to provide conclusions that are facts rather than interpretations. Equation (9), known as the structure factor, allows the amplitude of each X-ray reflection from a lattice to be calculated from the contents of the unit cell.

(9) $$F\left(h,k,l\right)=\overset{N}{\underset{j=1}{\sum }}F_{j}e^{2\pi i\left(hxj+kyj+lzj\right)}$$

*F*(*h*, *k*, *l*) is the amplitude of a particular reflection with indices (*h*, *k*, *l*) from mass at fractional unit cell coordinates of (*x_j_*, *y_j_*, *z_j_*) and with scattering factor of *F_j_*.

Equation (9) can be simplified for the 2D myofilament lattice (the 3D lattice projected down the filament long axis) to give the contribution of the actin and myosin scattering factors to each equatorial reflection, considered as simple rotationally symmetric densities (i.e., roughly cylindrical structures viewed down their long axes). In the case of the muscle A-band lattice, the unit cell contains one myosin filament at coordinates (0, 0, 0) and two actin filaments at (1/3, 2/3, 0) and (2/3, 1/3, 0). This gives a general expression for the amplitude of a muscle equatorial reflection as in Equation (10):

(10) $$F\left(h,k,0\right)=F_{M}+F_{A}\left(e^{2\pi i\left(\frac{h}{3}+\frac{2k}{3}\right)}+e^{2\pi i\left(\frac{2h}{3}+\frac{k}{3}\right)}\right)$$

Here, *F_M_* and *F_A_* are the scattering factors of the myosin and actin filaments respectively in those particular directions. By calculating the contribution of the myosin and actin scattering factors to each equatorial reflection it is possible to deduce possible reasons why the changes in intensity described above occur. The reasoning is based upon evidence from electron density maps of the A-band lattice using Fourier synthesis, and time-courses of mass transfer in the 9 nm annulus around the thin filament centres during contraction [15,23,79,82,83,84]. These data indicate that as muscles contract, mass from around the myosin filament centres moves away from the thick filaments towards the thin filaments. This moving mass is thought to be the myosin cross-bridges as they attach to the thin filaments during contraction. Table 2 shows the calculations of the multiplication factor of *F_A_* in Equation (10) for each equatorial reflection (*e*^2π*iθ*^ has been converted to cos (2π*θ*) + *i*sin (2π*θ*) for the purpose of the calculations).

As shown in Table 3, based on calculations of the simple model using Equation (10), each reflection out to the A(30) is observed to change in intensity in a way consistent with the general movement of cross-bridge mass to actin during the rising phase of the tetanus. If the myosin heads just moved out radially without clustering around actin the result would be different. In particular the A(11) peaks would not increase in such a marked way, as in rigor muscle where we know the heads are attached. The characteristic of equatorial X-ray diffraction patterns from rigor muscle, where most heads are actin-attached [85,86], is to have a relatively strong A(11) peak and a relatively weak A(10) peak, consistent with the analysis in Table 3. Active muscle has intensities part way between the resting and rigor values. What is very different here, is that the outer reflections A(31), A(40) and A(32) change in a different way to that expected, indicating more complexity in the cross-bridge cycle than in the simple model used to calculate Table 3, where the heads are just assumed to add mass to the actin positions [87,88].

An indication of what this added complexity might be comes from the observation that not only does the A(11) intensity still lead tension and have a different shape to its time-course than the tension, even with partial SL control, but other weaker peaks like the A(30), A(31) and A(32) peaks all change very rapidly and overshoot their plateau values, in the case of the A(30) and A(32) peaks roughly by a factor of two, before the tension plateau is reached. Assuming (as above) that the observed intensity changes are solely due to myosin head movements, the time-courses of the equatorial peaks in Figure 4 and Figure 5, particularly the higher orders in Figure 6, show that there is not just a change in the number of the attached heads, but that at an early stage in the development of tension there are heads in a different conformation or distribution of conformations to when the full tension (plateau) level is reached. This is unambiguous.

In thinking about what this might mean, we believe [41] as do others that active muscle contains a mixture of at least three myosin head structural states; some strongly attached heads (AM.ADP.Pi, AM.ADP, AM), some detached, resetting heads (M.ATP to M.ADP.Pi) and thirdly some heads in a state (AM.ADP.Pi*; possibly weak binding, pre-powerstroke heads) which would give a relatively strong A(11) peak, an A(10) not far from its relaxed value and strong outer intensities at the A(30), A(31) and A(32) positions. This latter state may be the non-force-producing initial actin-attached state in fish muscle as suggested by Harford and Squire [15]. It may be the elusive pre-powerstroke state that is often discussed in muscle cross-bridge scenarios [9,19].

### 4.3. Modelling the Equatorial Time-Courses

In an attempt to model objectively the equatorial time-courses we have supposed that what is being observed is a mixture of structurally different states. We assume that the myosin and actin filament backbones are virtually unchanged throughout a tetanus at the resolution we are considering (60 Å at best) and that all the observed changes are due to crossbridge movements and crossbridge shape changes.

**Figure 7:**
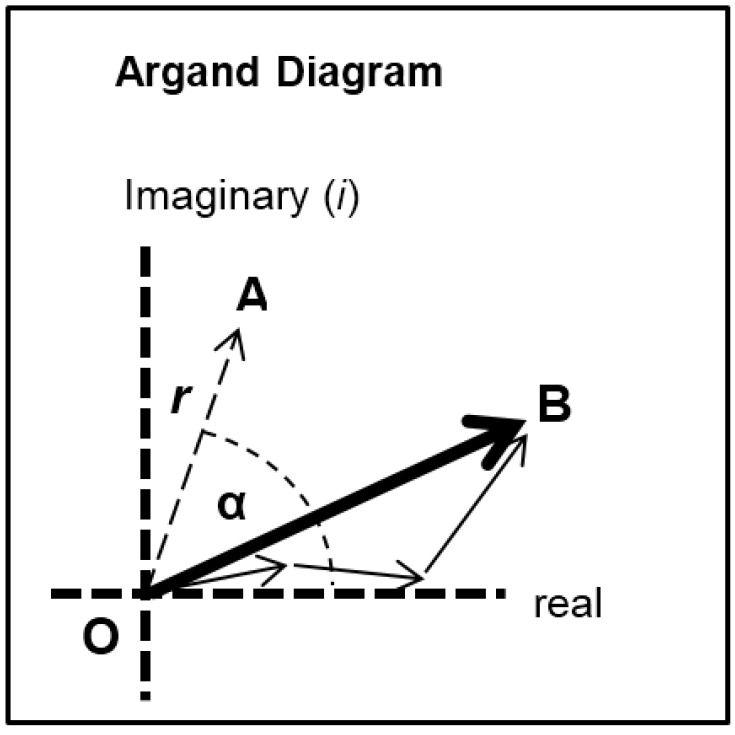
Diagram illustrating the addition of vectors in an Argand diagram. Here any vector such as OA has an amplitude (the arrow length) and a phase (α). In the muscle case for each equatorial reflection each crossbridge state will contribute a vector (the smaller arrows) which will add vectorially to give the resultant OB. If the structure is centrosymmetric then the arrows will all point along the horizontal (real) axis (their phase is 0° or 180°) and they just need to be added or subtracted.

Each of the different structural states will make a specific contribution to the diffraction pattern. Let us consider some of the equatorial peaks. Each reflection will be described by the structure factor *F*(*h*, *k*, *l*) as in Equation (9). This is a complex quantity described by an amplitude (square root of the observed intensity) and a relative phase. This can be shown as a vector on an Argand diagram as in Figure 7. For an arbitrary peak, the length OA in Figure 7 represents the amplitude and the angle α with the horizontal axis represents the phase. If there is substitutional replacement of one myosin head state for another, then the structure factors for each state add vectorially as in Figure 7 where each small arrow represents the contribution of a different state. In order to find the total resultant amplitude (OB), one needs to know the amplitudes and phases from each state and then to add them vectorially as in Figure 7 to get the resultant OB. However, in muscle at low resolution, the muscle structure projected down the filament long axis, which the equatorial peaks tell us about, is likely to be approximately centrosymmetric. This means that for every projected density at *x*, *y* there is an identical density at −*x*, −*y*. The effect of this on the structure factor is that the phases (α) of the equatorial peaks, rather than being anywhere between 0° and 360°, can only be 0° or 180°. The corresponding vectors in Figure 7 lie along the horizontal line (the real axis) through the origin and adding vectors is a relatively trivial exercise; the amplitudes are either added or subtracted along that line. Observations of the equatorial time-courses also suggest that the phases of the peaks do not change during the contraction; none of the amplitudes drops close to zero and then comes up again as might be expected if the contribution from one of the structures in the crossbridge cycle had a different phase (i.e., 0° instead of 180°).

The logic of the analysis is that: (i) there might be several crossbridge states present during the rising phase of the tetanus; (ii) that each would have its own particular contribution to the observed diffraction pattern, depending on the particular structure and how many crossbridges are in that state; and (iii) that the observed amplitudes at low resolution will be a simple sum of the amplitudes (positive or negative) from each structural state. Because of this we set up a computer model consisting of several structural states with forward and backward rate constants between each (Figure 2a). We allowed the heads to start in a structure like resting muscle and to enter a cycle after activation and an appropriate delay (the latent period); the tension and the observed equatorial peaks do not change instantly when activation occurs—there is a delay (Figure 4). With this model established we could define the structure amplitudes for some of the states (e.g., relaxed) and we could allow the computer program to use simulated annealing (see Methods) to search over other amplitude values (between limits) to give an optimal fit between the observed and calculated time-courses.

A factor to take into account in this kind of analysis is the number of observations being modelled and the number of parameters being searched over. Clearly the number of unknown parameters must be significantly less than the number of independent observations to give a reliable result. As we detail elsewhere [88], one reason that the modelling of the interference effects on the meridian was not successful, as discussed in the introduction, was that the number of parameters being modelled was significantly more than the number of observations; the problem was under-determined.

Here we are modelling the time-courses of eight equatorial peaks and we estimate that each time-course contains at least four, possibly five, observations (the number of parameters required to model the time-course as a polynomial/exponential combination). Therefore there would be 32 to 40 observations. In parameter searches to find the crossbridge states, we need to keep the number of model parameters rather less than about 32. Table 4 shows the results of this modelling.

#### 4.3.1. Two-State Models

**Figure 8:**
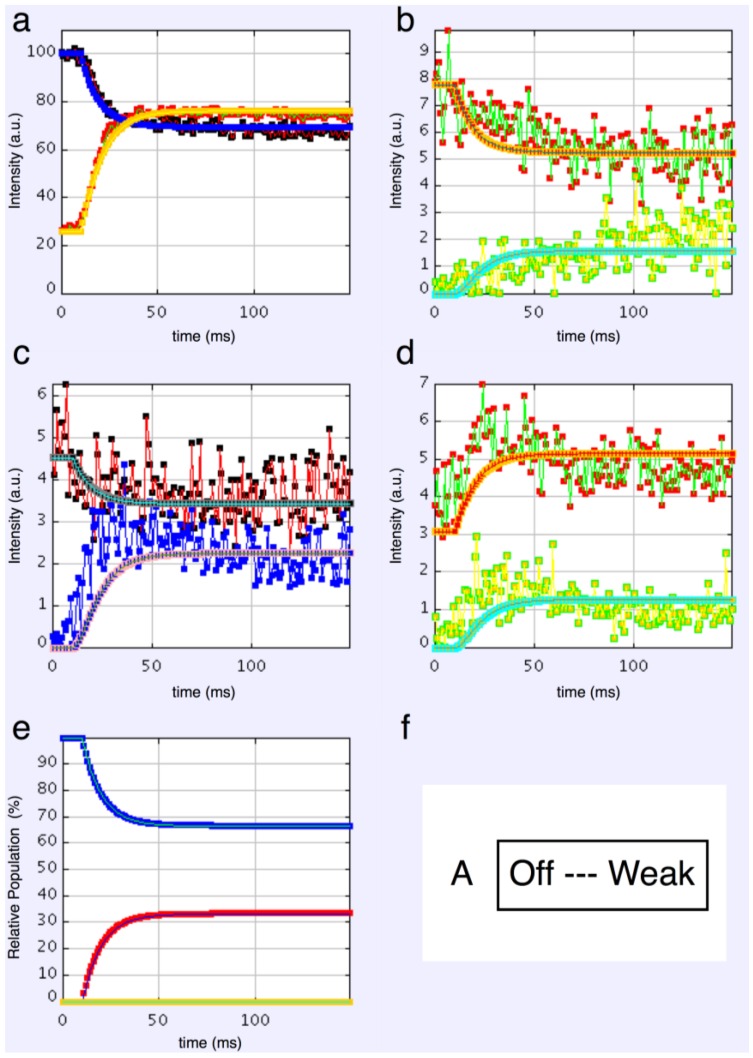
Plots from simulated annealing fits of the 2-state model (A in Figure 2b) to the observed equatorial time-courses in Figure 4 and Figure 6, with SL control applied. The fit is reasonable (Chi = 1457), but there are clearly no overshoots in any of the time-courses. (**a**) Plot of the A(10) data, in black, and the fit, in blue; the A(11) data, in red, and the fit, in yellow; (**b**) Plot of the A(20), in red, and the fit, in orange; the A(40) data, in green, and the fit, in turquoise; (**c**) Plot of the A(21) data, in black, and the fit, in pale blue; the A(31) data, in bright blue, and the fit, in purple; (**d**) Plot of the A(30) data, in red, and the fit, in orange; the A(32) data, in green, and the fit, in turquoise; (**e**) Plot of the populations in the two states: off is blue, weak is red. The populations at the active plateau are 67% off and 33% weak (Table 4); (**f**) The two states in model A: off and weak.

We started with simple 2-state models (Models A and B in Figure 2b). The heads were either in the relaxed (off) state, or were attached and all in the same conformation. For Model A (off and weak), the number of parameters involved was: 2 rate constants, 1 delay (the latent period), and the eight unknown amplitudes of the attached structural state (11 parameters to model). The results from this very simple model were reasonable, Figure 8, Chi = 1457 (Table 4). Note that the modelling also gives the time-course of the changing populations of the states involved.

Here the steady state values for the active tetanus plateau were 67% detached and 33% attached. The latent period was about 10 ms. In the attached state the amplitudes are not typical of relaxed or rigor patterns; the best attached state had a very strong 11 peak (Table 5). However, it is clear from such a simple scheme that none of the peaks will overshoot as observed—at least one intermediate attached state is needed to generate an overshoot. One can also do this calculation as a 2-state model which simply has relaxed heads as one state and rigor heads as the attached state (Model B in Figure 2b). The agreement here was very poor indeed (Chi = 7802: Table 4); the active structure is not a simple mix of relaxed and rigor heads.

#### 4.3.2. Three-State Models

**Figure 9:**
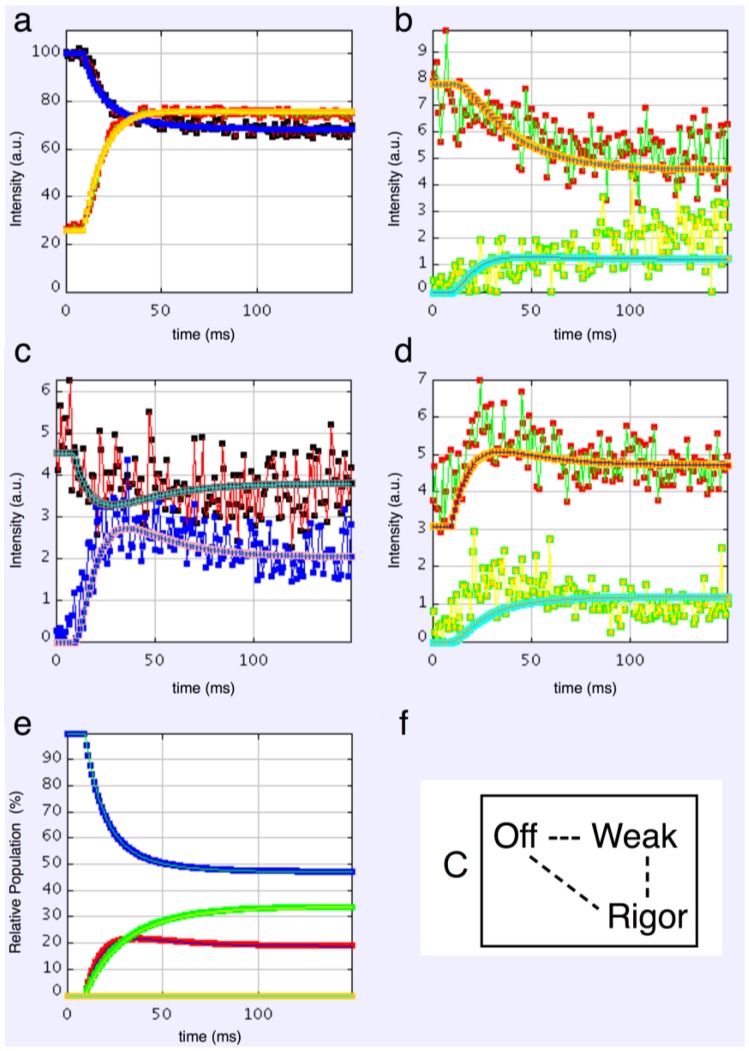
Plots from simulated annealing fits of the 3-state model (C in Figure 2b) to the observed equatorial time-courses in Figure 4 and Figure 6, with SL control applied and the rigor 20 allowed to be freely fitted. The fit is good (Chi = 1240 (Table 4)), and this time there is very clear evidence of the overshoots being modelled. (**a**) Plot of the A(10) data, in black, and the fit, in blue; the A(11) data, in red, and the fit, in yellow; (**b**) Plot of the A(20) data, in red, and the fit, in orange; the A(40) data, in green, and the fit, in turquoise; (**c**) Plot of the A(21) data, in black, and the fit, in pale blue; the A(31) data, in bright blue, and the fit, in purple; (**d**) Plot of the A(30) data, in red, and the fit, in orange; the A(32) data, in green, and the fit, in turquoise; (**e**) Plot of the populations in the three states: off is blue, weak is red and rigor-like is green. The populations at the active plateau are 48% off, 20% weak and 32% rigor-like (Table 4); (**f**) The three states in model C: off, weak and rigor-like.

3-state models involved starting from the relaxed state and then having two actin-attached states (Model C & D in Figure 2b). It might be assumed (Model C) from the Lymn-Taylor scheme [3] that one attached state (the strong state in Figure 2) might possibly be like the rigor state and we know the equatorial intensities from rigor fish muscle at least out to the 30 peak (see Supplementary Materials Figure S14). The weak state is unknown. On this basis the parameters involved are six rate constants, a delay, the eight amplitudes for the non-rigor (weak) attached state and the three outer rigor peaks (19 parameters to model). The results from this are quite good. However since we know that the 20 has a contribution from the Z-line (the Z11), the results were substantially improved if the A(20) in the rigor state was a free parameter. The final result in Figure 9 was very good agreement, with as Chi of 1240.

If the strong structural state in Figure 2b is not rigor (Model D), then the amplitudes from both of the two attached states are unknown. Parameters to find are six rate constants, one delay, and the eight amplitude parameters for each of the two attached states (23 parameters total). The best fits in this case were little different from the analysis with rigor in the cycle.

#### 4.3.3. Four-State Models

At the risk of having too many unknown parameters, a fourth state can be included into the cycle. It is termed reset in Figure 2a, but it could be either the first detached state of the heads prior to the hydrolysis step (Model E in Figure 2b) or a third actin attached state (Model G in Figure 2b). Is there any merit in including this fourth state? If one of the attached states is like rigor and the other two are unknown, then there are 28 parameters to fit. In this case the best Chi was 1270 (Table 4). If the rigor state is not present and there are three unknown structures (Model F in Figure 2b) then there are 33 parameters (really rather too many) and the best Chi was 1298 (Table 4), not significantly better than other Models.

#### 4.3.4. Modelling Summary

**Figure 10:**
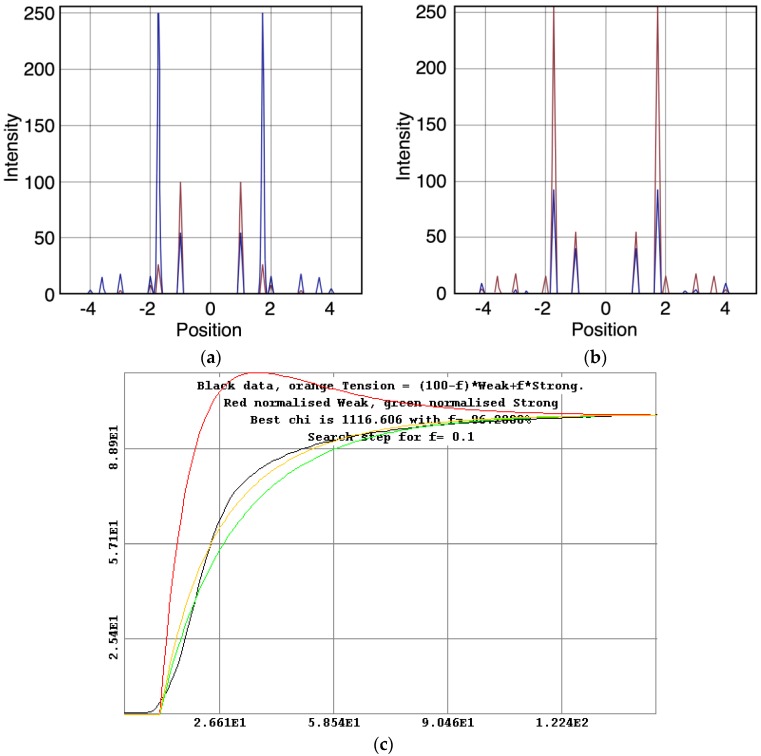
Plots of the best modelled X-ray amplitudes, tension and state populations for the 3-state model (C in Figure 2b) shown in Figure 9. (**a**) Modelled X-ray amplitudes for the off state, in red, and the weak state, in blue; (**b**) Modelled X-ray amplitudes for the weak state, in red and the rigor-like state, in blue; (**c**) Plot of the tension calculation for the 3-state model including rigor in which a percentage f is in the strong state and a percentage (100 − *f*) is in the weak state. The experimentally measured tension is shown in black, the fitted tension in orange and the noramlised populations in the weak and rigor-like states in red and green respectively. The fitting gives most heads in the rigor state (*f* = 96%).

In summary, the results of the time-course fitting are as follows:

(1) The observed higher order reflection time-courses can be modelled quite well.
(2) There must be more than two states to explain the observed overshoot of the A(11) and particularly the higher order A(30) and A(31) peaks.
(3) Despite the rapidly increasing number of parameters to fit, there is no compelling reason to go from three to four states. The fit only changes marginally on going from 19 to 28 parameters or 23 to 33 parameters (Table 4), where one might expect a substantially better fit if the extra step is important.
(4) Including rigor as one of the attached states gives as good results as anything else.
(5) The “weak” state that needs to be added, whether with 2-, 3- or 4-state models, always has the same sort of transform. The A(11) and other intensities in the rigor pattern are not enough to give the observed overshoots. The weak state (Table 5; Figure 10a,b) has an exceptionally strong A(11) reflection.

We have assumed that the structure is approximately centrosymmetric, giving equatorial phases of 0° or 180° only. Vertebrate striated muscle myosin filaments themselves have a somewhat 3-fold structure as shown by AL-Khayat et al. [89], but the crossbridge arrays in the two halves of a single thick filament will have opposite hands when projected down the filament axis and there is also a roughly 40° rotation between equivalent crowns on each side of the bare zone, Luther et al. [48].

Thus, at the resolution that we are considering the thick filaments are likely to appear approximately centrosymmetric in projection. However, note, finally, that even if the phases are off by a few degrees from 0° to 180°, this calculation is still valid. For example, a 5° error in each phase would in the worst case scenario change the sum of the amplitudes of a particular peak (OB in Figure 7) in the 4-state model from 4 × *R* to 4 × cos(5) × *R* = 3.985*R*, where *R* is the amplitude from each state; a change of 0.375%. (Note that in the worst case scenario the Rs from the different states would all be the same). An average 10° phase error would in the worst case give 3.939R instead of 4, an error of 1.5% in OB. A 5% error in OB would arise from an average worstcase phase error of 18.2°. If there are fewer states then the error would be proportionately less.

With the modelling completed and the conclusion reached that a 3-state model including rigor is as good as anything in explaining the observations, there remain some obvious questions: (i) What is the “weak” state like? (ii) What are the rate constants around the cycle? (iii) What are the populations of the attached heads at the tetanus plateau? (iv) How much tension is generated by heads in each of the different attached structural states?

### 4.4. The Weak State

What is immediately apparent from Table 5 is that, whatever model is chosen, in order to generate the observed higher order overshoot, the diffraction pattern from the weak state is always pretty much the same. Table 5 compares the best modelled amplitudes from all of the Models that include a weak state, takes the average and calculates the standard deviation.

The consistency throughout is notable, so what is this state? The known structure with the nearest diffraction pattern to this is the weak binding state induced in rabbit psoas muscle fibres at low ionic strength (20 mM) observed by Brenner et al. [64]. This state was characterised by them and in mechanical studies by, for example, Shoenberg [90] as a relaxed state in the sense that ATP was present, but the muscle was not activated, and yet the heads were undergoing very rapid attachment-detachment steps such that the muscle stiffness rose rapidly from being low in relaxed and normal ionic strength fibres to gradually higher as the ionic strength was lowered if the stretches used to measure stiffness were fast enough.

In our case, the ionic strength is normal, but a state similar to the rapid equilibrium state of Brenner et al. [64] appears to be present, indicating that such a state may be part of the normal contractile cycle of myosin heads on actin that increases in abundance when the muscle is activated.

### 4.5. The Rate Constants

The rate constants in the best models are summarised in Table 4. For our preferred 3-state model including rigor, the off to weak rate constants, *k*_1_ and *r*_1_, are both quite large at 29.7 and 66.3 per second, consistent with a rapid equilibrium as in the weak-binding state. The transition from weak to strong structural states is a much slower process with *k*_2_ at 7.5 and *r*_2_ insignificant. The strong to off state is faster once again with *k*_5_ and *r*_5_ being 17.2 and 28.2, respectively. Although we are looking at structural states, which do not necessarily relate to different biochemical states (see later discussion), it appears that the rate limiting step is the weak to strong (force-producing) transition, as might be expected in an isometric contraction.

Of course, it is quite likely that some of the steps we are considering have rate constants that are strain-dependent. What we are modelling are the best simple rate constants to generate the observed variations in amplitude of the X-ray reflections. The fit is good, suggesting that the strain-dependence might not be too great in the quasi-equilibrium situation found in isometric contractions.

### 4.6. The Population of States

The modelling necessarily includes the time-courses of the populations of the different states. These populations are summarised in Table 4. For the 2-state model not including rigor the two populations are 67% off and 33% attached. For the more realistic 3-state model including rigor, the off population is 48%, the weak population 20% and the rigor population 32%. However, as discussed below, these populations should not be taken to be the proportions in different biochemical states. It is interesting to note that the results from the paper of Wu et al. [91] using a totally different approach with insect flight muscle tomograms came to the conclusion that 53% of the heads bind to actin with 29% in the strong conformation, which is very similar to our own conclusions.

### 4.7. The Tension Distribution and the Crossbridge Mechanism

Since we know the time-course of the change in isometric tension and we know the time-courses and populations of the various states, it is possible to assign to each state a parameter (i.e., percentage *f*) representing the tension associated with that state and then to optimise the sum of the individual state tensions against the observed total tension to find out how much each state contributes. Figure 10c shows this for the 3-state model including rigor (Model C in Figure 2b). Here the best fit is obtained as *f* × strong and (100 − *f*) × weak. The best fit to the observed tension is obtained if the rigor state provides (*f* =) 96% of the tension and the weak state (100 − *f* =) 4%, after a very clear overshoot in its time-course.

**Figure 11:**
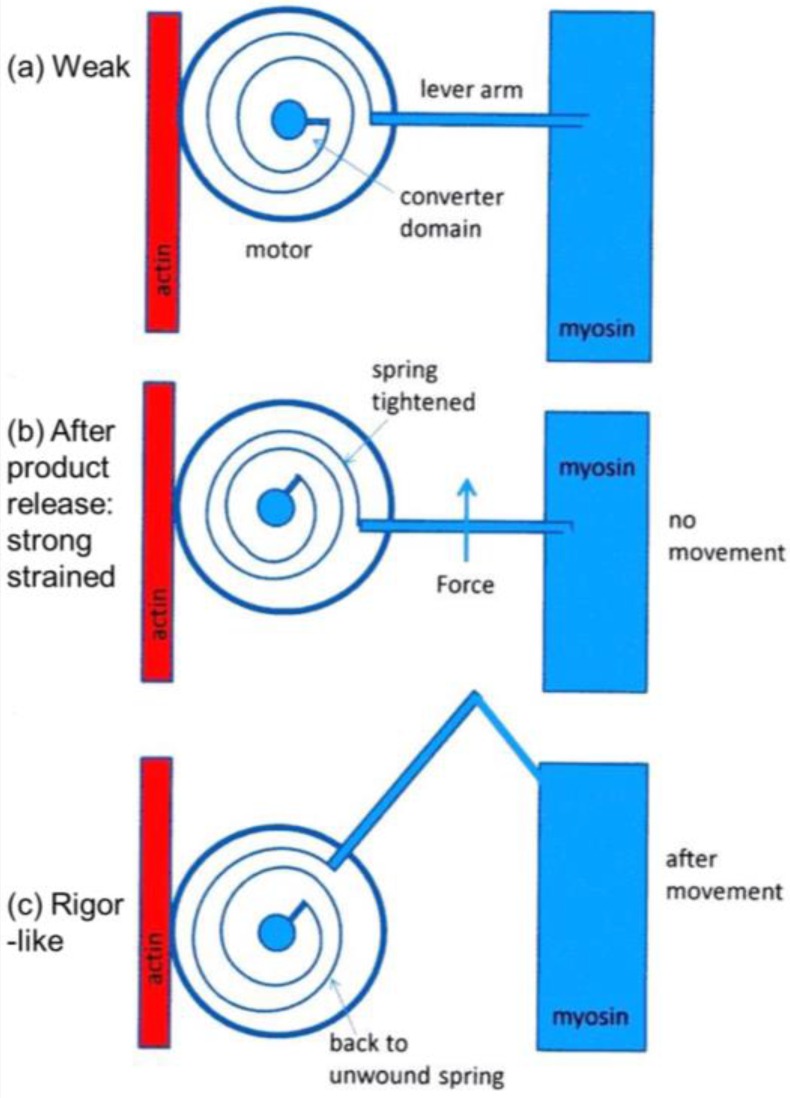
Schematic diagram to show how a crossbridge might behave if the crossbridge compliance is within the motor/converter domains. The compliant element is visualised as a spiral spring which is unstrained in (**a**); which might correspond to initial, weak, attachment as AM.ADP.Pi. With the release of products Pi and ADP the effect is to tighten the spiral spring (**b**); but if the filaments unable to move, the lever arm would stay in the same position as (**a**); but would be strained. Only if the filaments can move would the lever arm rotate, thus relieving the strain in the spring (**c**).

It was mentioned earlier that the structural states that we see are not necessarily correlated one-to-one with the known biochemical states in the crossbridge cycle. The reason for this can be seen in the kind of crossbridge mechanism illustrated schematically in Figure 11. Here the myosin head is depicted as having elasticity (compliance) in the form of an internal spiral spring. In the weak state (Figure 11a) the spring is assumed to be in its unstressed position. This would correspond to the weak state AM.ADP.Pi. With the release of products (Pi and perhaps ADP), the resulting internal structural rearrangement in the head would tighten the spring (Figure 11b) to generate force. However, at this point, the lever arm need not be in a different place from where it is in the weak binding state in Figure 11a. It will exert force between the filaments, but may not have moved. It will be a strained strong state. If the elasticity of the myosin is all in the head and not in S2, then the lever arm will only move if there is relative axial movement of the filaments (Figure 11c), ending in the rigor conformation of the lever arm. If there is filament sliding and lever arm rotation, then the internal spring would gradually become unwound again, until at the end of the stroke the force between the filaments is zero.

**Figure 12:**
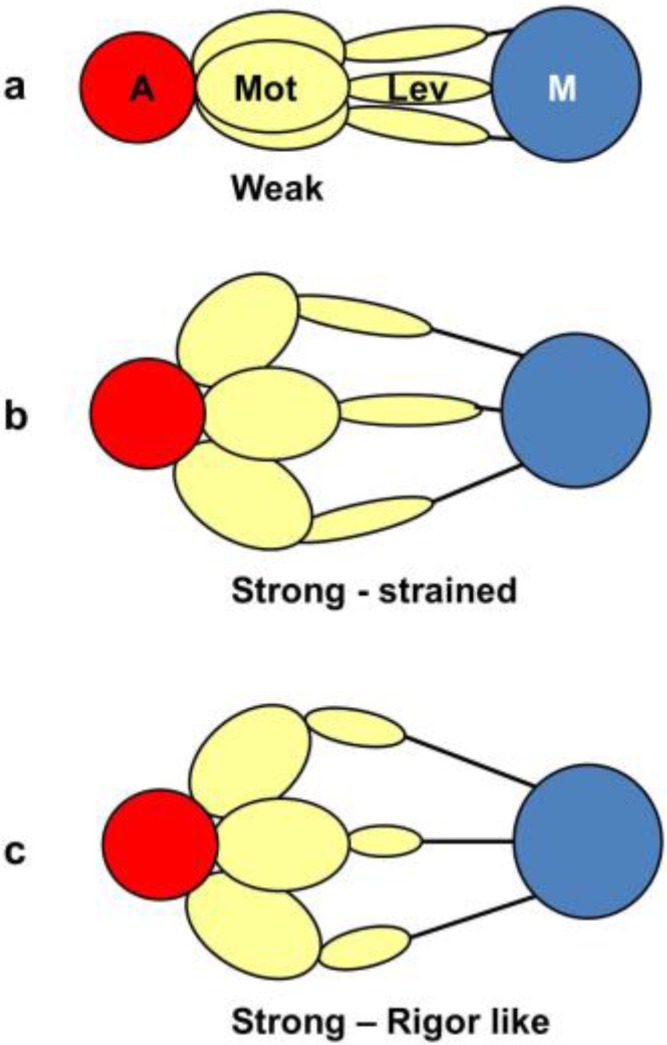
The equivalent schematic transitions to Figure 11, but this time as viewed down the fibre axis, i.e., as it would appear on the equator. The weak binding state AM.ADP.Pi would presumably have the motor domains in a loose (non-stereospecific) attachment to actin (**a**); so the heads would point back to their origins on myosin. The release of products would require the motor domains to become stereospecifically bound to actin, so they would follow the actin helix and become more spread azimuthally (**b**); The heads would still be axially strained as in Figure 11b. Relative filament sliding as in Figure 11c would relieve the strain by allowing the lever arms to tilt (**c**) and the strain would gradually reduce to zero. On the equator, configurations (**b**,**c**) would probably appear rather similar because of the relatively low mass of the lever arm. In other words, the modelled rigor-like state would include both (**b**,**c**).

From a side view, as in a longitudinal section, the weak state in our modelling would appear as a mixture of the states in Figure 11a,b. We would expect some of those heads not to produce force (Figure 11a) and some to produce force after product release (Figure 11b) and these two states might look the same when viewed from the side as in Figure 11. However, we are looking at the equator of the pattern; the view of the structure projected down the fibre axis. What would happen there? Figure 12 shows what might happen in projection down the axis. We would imagine that a weak state would be characterised by heads that attach non-stereospecifically to actin in such a way that they all point back to where they came from on myosin. In this way they would be lying along the 11 planes in the lattice and would give the observed strong A(11) reflection.

At some point in the cycle, after release of products, the heads would become stereospecifically attached to actin and because of the varying azimuths of the actin monomers the initial strongly bound heads would become more azimuthally distributed around the actin filament axis (Figure 12b), no longer all pointing along the 11 planes. Thus, the A(11) reflection would be weaker, as in the rigor pattern. Filament movement and lever arm swinging would then result in a foreshortened lever arm in projection down the filament axis (Figure 12c), but this would make little difference to the equatorial diffraction pattern. In other words, the states in Figure 12b,c would look rather similar on the equator because of the relatively small mass of the lever arm compared to the motor domain. As discussed in the Introduction, we previously [41] came to the same conclusion that the lever arm would make little contribution to the meridional diffraction pattern at low resolution because of its small mass.

What we observe as the rigor state will be a mixture of the initial stereospecifically-bound but strained high tension state in Figure 11b, but viewed down the axis (Figure 12b) and the final rigor-like (low tension) state (Figure 11c and Figure 12c) and everything in between. Thus, our results show for the 3-state model (C in Figure 2b) that 48% + 20% of the heads are in the off and weak-binding states, that 32% of the heads are in the first stereospecific and rigor-like states and that these 32% of heads together produce about 96% of the tension. Note that in our previous analysis of the meridional diffraction pattern [41] and the effects of length steps, we also concluded that the motor domains, which dominate the diffraction patterns, would have 70% (off and weak) not moving with the step and 30% (strong) moving as the filaments move. The two sets of results are closely compatible.

## 5. Conclusions

We have shown that the Plaice fin muscle is a viable preparation to use for recording time-resolved low-angle X-ray diffraction pattern from active whole muscle with sarcomere length control. Suitable equipment for recording and control of SL has been developed and used successfully. The tension in the rising phase of end-held tetanic contractions and the intensities of the many of the equatorial reflections all change much faster with partial SL control than without, but there is still evidence for a myosin head state at an early stage of the rising phase which has the characteristics of rapid equilibrium actin attachment, but where the force level is still low (see also Tanaka et al. [92]). We have shown that: (i) a 3-state model including rigor fits the data as well as any other model; (ii) there is no need to have more than two structurally distinct attached states when viewed on the equator, although each state may include more than one biochemical state; (iii) we have defined the relative populations of states in this model; and (iv) we have determined the contribution that the structural states make to the total tension.

The analysis done here tests whether the observed changing equatorial intensities can be modelled as a sum of known or unknown contributions. If they are unknown it defines what these might be. One of the known patterns is that of rigor muscle and this seems to work pretty well as part of the cycle. We go on to discuss what the results might mean in terms of models in Figure 11 and Figure 12. What these show is that the rigor state that we are modelling appears to be showing the conformation and azimuthal distribution of the motor domains of the attached heads in the strong state. The lever arms in this scheme can be tilted away from the rigor conformation (Figure 11b) where they would produce tension, or have tilted to the rigor position (Figure 11c) where no tension would be produced. Both of these head conformations would look similar on the part of the equator that we are studying because of the relatively low mass of the lever arm.

We are now in a position to start to analyse the whole recorded 2D diffraction pattern in more detail in order to further refine myosin head behaviour in active fish muscle.

## Figures and Tables

**Table 1:** Comparison of times for 50% change (T_1/2_) during the rising phase of tetanic contractions in various example intact muscles, including plaice fin muscle in the present study.

Animal and Preparation Type	T (°C)	T_1/2_ Tension (ms)	T_1/2_I_10_ (ms)	T_1/2_I_11_ (ms)	Lead over Tension (ms)	Source
Frog	5–6	38 ± 4		27 ± 5	~10	Kress et al. [81]
Intact muscle						
Frog	4	49.4 ± 11.4	33.2 ± 16.6	27.6 ± 12.1	Of I_10_: 16	Cecchi et al. [69]
Intact fibre, fixed end					Of I_11_: 22	
Frog	4	45.4 ± 16.1	27.0 ± 10.7	23.4 ± 11.5	Of I_10_: 18	Cecchi et al. [69]
Intact fibre, SL clamp					Of I_11_: 22	
Turbot	5–8	41 ± 3	35 ± 8	21 ± 4	Of I_10_: 6	Harford and Squire [15]
Whole muscle, fixed end					Of I_11_: 20	
Plaice	7–8	33.8 ± 0.6	25.0 ± 3.4	20.5 ± 2.6	Of I_10_: 8.8	Present study
Whole muscle, fixed end					Of I_11_: 13.3	
Plaice	7–8	22.5 ± 0.8	19.5 ± 2.0	17.0 ± 1.1	Of I_10_: 3.0	Present study
Whole muscle, SL controlled					Of I_11_: 5.5	

**Table 2:** Structure factor calculations in Equation (10).

*h*	*k*	Cos(2π(*h*/3 + 2*k*/3))	Sin(2π(*h*/3 + 2*k*/3)	Cos(2π(2*h*/3 + *k*/3))	Sin(2π(2*h*/3 + *k*/3)	Sum of Cos Terms	Sum of Sin Terms	*F_A_* Factor
1	0	−0.50	0.87	−0.50	−0.87	−1.00	0.00	−1.00
1	1	1.00	0.00	1.00	0.00	2.00	0.00	2.00
2	0	−0.50	−0.87	−0.50	0.87	−1.00	0.00	−1.00
2	1	−0.50	0.87	−0.50	−0.87	−1.00	0.00	−1.00
3	0	1.00	0.00	1.00	0.00	2.00	0.00	2.00
3	1	−0.50	−0.87	−0.50	0.87	−1.00	0.00	−1.00
4	0	−0.50	0.87	−0.50	−0.87	−1.00	0.00	−1.00
3	2	−0.50	0.87	−0.50	−0.87	−1.00	0.00	−1.00

**Table 3:** Intensity changes expected on the equatorials with a simple model as in Equation (10), compared to what is observed.

Equatorial Reflection	*F*(*h*, *k*, *l*)	Estimated Intensity Change	Observed Intensity Change
A(10)	F_M_ − F_A_	Down	Down
A(11)	F_M_ + 2F_A_	Up	Up
A(20)	F_M_ − F_A_	Down	Down
A(21)	F_M_ − F_A_	Down	Down
A(30)	F_M_ + 2F_A_	Up	Up
A(31)	F_M_ − F_A_	Down	Up
A(40)	F_M_ − F_A_	Down	Up
A(32)	F_M_ − F_A_	Down	Up

**Table 4:** Results on the rate constants and state populations from the equatorial modelling for the cycles shown in Figure 2b. The best time-resolved values for all combinations are shown. Chi has an arbitrary scaling. Where “inc Rigor” is used it indicates the strong state used was the rigor state. “Rigor 20 free” indicates that all rigor reflections except the A(20) were allocated their observed values, whereas the A(20) was allowed to change between specified limits. *r*_6_ and *k*_6_ in Figure 2a were zero for these particular models.

Model Figure 2b	Model	Parameter No.	Lowest Chi	Delay (ms)	k_1_	r_1_	k_2_	r_2_	k_3_	r_3_	k_4_	r_4_	k_5_	r_5_	Populations (%)			
															Off	Weak	Strong	Reset
**A**	**2-state Free**	11	1457	10	34.0	67.9									67	33		
**B**	**2-state inc Rigor**	8	7802	19									107.0	52.7	32		68	
**C**	**3-state inc Rigor (Rigor20 free)**	19	1240	10	29.7	66.3	7.5						17.2	28.2	48	20	32	
**D**	**3-state Str Free**	23	1344	9	26.2	40.7	3.5	2.4					10.4	12.6	43	30	27	
**E**	**4-state inc Rigor**	28	1270	10	30.0	61.8	3.0	7.4	2.2	0.2	29.1	9.3			52	24	7	17
**F**	**4-state Str Free**	33	1298	9	23.6	47.9	5.3	2.4	0.4	4.0	5.0	4.7			40	20	20	20
**G**	**4-state inc Rigor**	28	1220	10	26.3	69.7	7.4	1.3	9.2	3.9	23.0	5.9			52	19	20	19

**Table 5:** Results on the best amplitudes from the equatorial modelling for the cycles shown in Figure 2b. Where “inc Rigor” has been used it indicates the strong state used was the rigor state.

State	Model	Reflections							
		A(10)	A(11)	A(20)	A(21)	A(30)	A(31)	A(40)	A(32)
Rest		10	5.1	2.79	2.13	1.76	0	0	0
Weak	2-state Free	5	16	1.3	1.3	3.3	4.5	3.4	3.8
	3-state inc Rigor	7.4	16	3.9	0	4.2	7.7	4.1	1.9
	3-state Free	6.3	16.8	2.4	2.1	5	5	3	1.7
	4-state inc Rigor (A)	6.4	18.3	2.3	0.9	4.6	5	1.5	2.7
	4-state inc Rigor (B)	5	19.2	0.5	1.5	4.9	7.6	5	4.6
	4-state Free	5	19.1	2.1	0.4	4.1	5.8	4.5	2
	Average	5.85	17.57	2.08	1.03	4.35	5.93	3.58	2.78
	SD	1.01	1.49	1.15	0.76	0.63	1.39	1.25	1.18
Middle	4-state inc Rigor (B)	7.8	6.8	0.0	0.0	0.10	0.20	0.60	3.70
Strong	2-state inc Rigor	6.32	9.61	4.81	2.8	1.62	1.8	1.2	1.5
	3-state Free	7.7	5.8	1.2	1.1	0	0	0.4	3.3
	3-state inc Rigor	6.32	9.61	4.81	2.8	1.62	1.1	1	5
	4-state Free	8.1	11.4	1.3	0	1.6	1.5	0	1
	4-state inc Rigor (A)	6.32	9.61	4.81	2.8	1.62	2	0.9	1.9
	4-state inc Rigor (B)	6.32	9.61	4.81	2.8	1.62	0	0.4	0.5
	Average	6.85	9.27	3.62	2.05	1.35	1.07	0.65	2.20
	SD	0.83	1.85	1.84	1.21	0.66	0.88	0.45	1.67
Reset	4-state inc Rigor (A)	6.2	5.1	0	2.8	0	0.8	4.5	2.8
	4-state Free	8	2.5	1.8	4.7	1.4	0	0	4.3
	Average	7.1	3.8	0.9	3.75	0.7	0.4	2.25	3.55
	SD	1.27	1.44	0.87	1.49	0.57	0.41	2.04	1.12

## Supplementary Materials

The following is available online at www.mdpi.com/2079-7737/5/4/41/s1. Document: Additional Materials and Methods.

## Abbreviations

The following abbreviations are used in this manuscript:

ADP	Adenosine DiPhosphate
ATP	Adenosine TriPhosphate
Pi	Inorganic Phosphate
2D	Two-Dimensional
SL	Sarcomere Length
