# Supplementary material for: X-ray Diffraction Evidence for Low Force Actin-Attached and Rigor-Like Cross-Bridges in the Contractile Cycle
Source: Biology (Basel). 2016 Oct 26;5(4):41. doi: 10.3390/biology5040041 (PMC5192421; doi:10.3390/biology5040041)
Supplement: Supplementary file 1 [file biology-05-00041-s001.zip › biology-149789-supplementary/biology-149789-supplementary-final.pdf]

Supplementary Materials: X-ray Diffraction Evidence for Low Force Actin-Attached and Rigor-Like Cross-Bridges in the Contractile Cycle

Felicity Eakins, Christian Pinali, Anthony Gleeson, Carlo Knupp and John M. Squire

1. Plaice and Turbot Muscle Dissection

Although the present paper is all on Plaice fin muscle, the dissection of turbot fin muscle which we use in other studies is similar and is included here (see Figure S1). Much of the dissection method was developed together with Dr. Jeffrey J. Harford, to whom we are indebted (see Harford [1]).

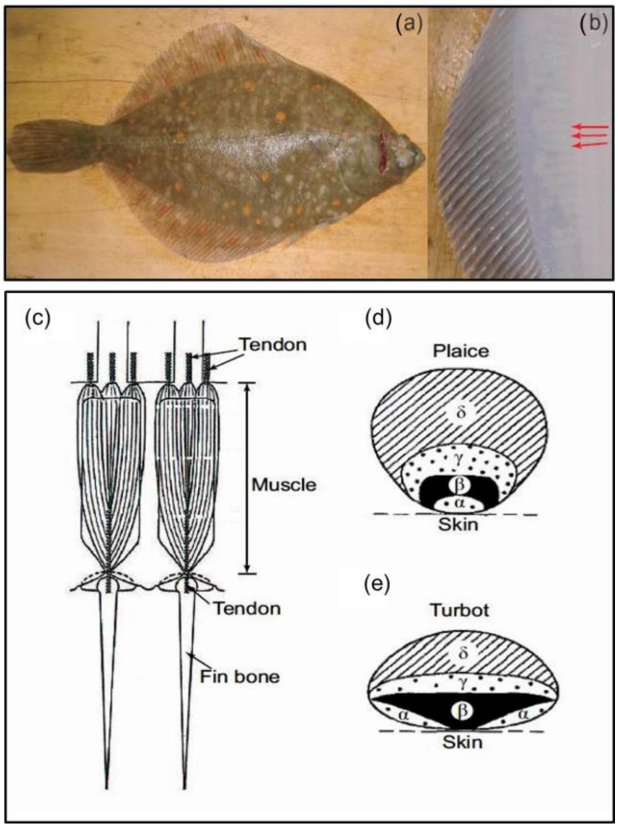

Figure S1. Photographs of: (**a**) *Pleuronectes platessa* or the Plaice; (**b**) examples of fin muscles used in this investigation, arrowed in red. These muscles are positioned just under the skin of the fish, having several tendons attached to them at one end, and at the other, an attachment to the fin bone by a single tendon. The pale underside of the fish is shown; (**c**) Diagram of the anatomy of Plaice fin muscles, although the situation is very similar in Turbot fin muscles which we have used previously. Two muscles are shown with the single tendon attachments to the fin bones at the distal end of the muscles and the multiple attachments to the underside of the skin at the proximal end. The splitting of the muscles into three or four lobes at the proximal end is also shown. Figure taken from Chayen et al. [2]; (**d**,**e**) Diagrams of the cross-sectional view through Plaice (**d**) and Turbot (**e**) fin muscles, perpendicular to the long muscle axis. The muscles can be divided into four blocks depending on fibre type, labelled α; β; γ; δ. The positions and fibre compositions of the four regions differ slightly between the two types of flatfish, but the majority of fibres in the plaice are fast (δ). Figures taken from Chayen et al. [3].

Flatfish were obtained from London University Marine Biological Station (U.M.B.S.), Isle of Cumbrae, Scotland and from Aquarium Technologies Ltd., Weymouth. The fish were kept in tanks of re-circulating sea water at 12–14 °C for no more than 2 months. For the synchrotron experiments,

the fish were kept in aerated tanks of sea water maintained at 5–7 °C for approximately one week. Flatfish were stunned by a blow to the head, and subsequently killed by destruction of the brain. Several small blocks of the dorsal and anal fin tissue and bones, approximately 5 or 6 muscles long, were removed from each fish (Figures S1–S5) and placed in aerated Ringer solution on ice. Two viable muscle preparations were usually obtained from each block of tissue.

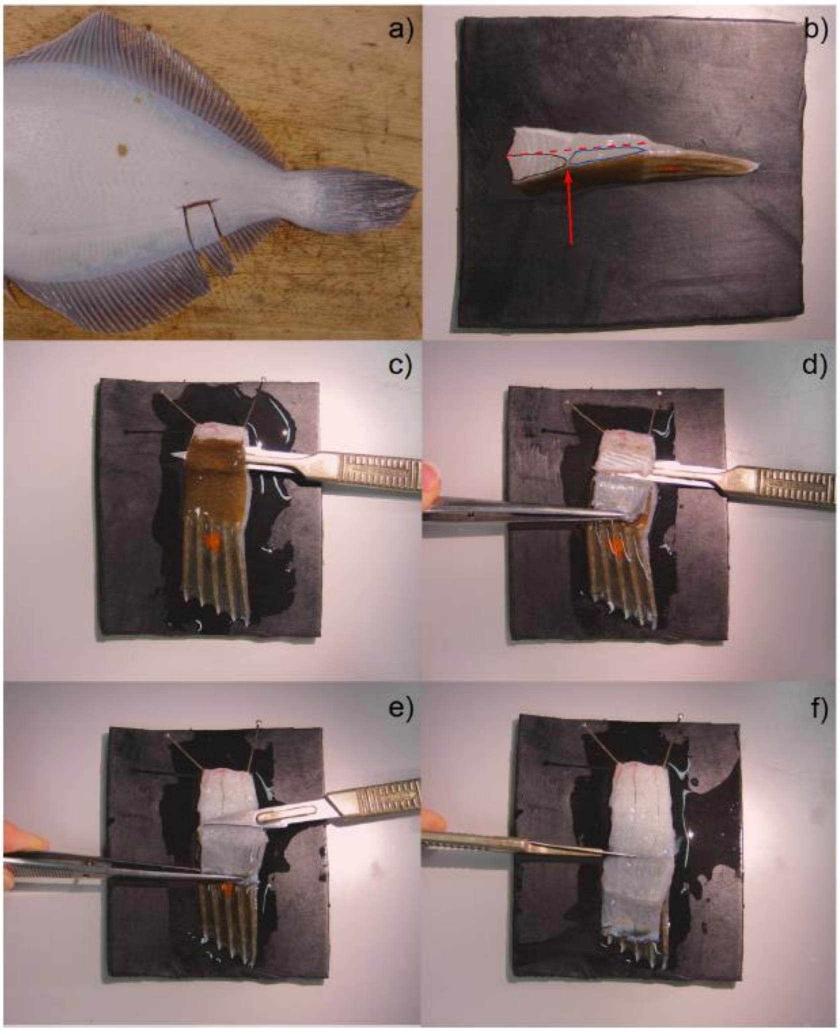

Figure S2. Photographs of: (**a**) a typical block of fin muscles used for fin muscle dissection; (**b**) side view of the tissue block showing the myotomal muscles outlined in black, the fin muscles outlined in blue and the central layer of bone in red; (**c**) procedure to remove the skin from the myotomal muscle; (**d**) removal of the myotomal tissue; (**e**) separation of the fin muscles from the central layer of bone; (**f**) removal of the central block of bone and the lower layer of fin muscles to leave just the fin muscles attached to the upper skin and fin bones.

Figure S2b shows the anatomy of the sample in cross-section through the fish. The area of fin muscles consists of four layers of muscles in two pairs. In each pair, one muscle layer abutts the skin and the other is next to the central block of bone. The first step in the dissection was to use two pins to tether the fin tissue to a piece of rubber, one pin through the pale under-skin of the fish and the other through the two outer fin bones of the block of tissue. The specimen was kept constantly cool and moist by pipetting fresh iced Ringer solution onto it at regular intervals during the dissection. A probe was used to loosen the gap between the myotomal and fin muscles, just below the upper layer of skin, arrowed in Figure S2b. A scalpel was inserted into the gap, sharp edge away from the fin bones, and the skin cut away from the myotomal muscle as shown in Figure S2c. The skin was pulled back and a scalpel slipped under the edge of the myotomal muscle, again with the edge facing away from the fin bones, taking care not to damage the fin muscle tendons connected to the underneath of

the skin at this point. Keeping the scalpel almost flat to the bone the myotomal tissue was cut away from the central block of bone and discarded, see Figure S2d. Pulling the skin back quite tightly, the scalpel was then positioned facing towards the fin bones, again almost flat to the central block of bone, and held firmly against it. By cutting very carefully from side to side, the fin muscles were cleared away from the central layer of bones, making sure the scalpel was always held firmly against the bone (Figure S2e). This ensured that only the layer of fin muscles next to the central raft of bone was damaged by the cutting. When the joints between the central bone layer and the fin bones were reached, the scalpel was used to cut straight down through the specimen, to leave just the fin bones and fin muscles attached to the upper layer of skin (see Figure S2f). Figure S3a shows the specimen pinned out with the upper skin face down. It illustrates the removal of the remnants of the layer of fin muscles which were next to the central bone layer from the fin muscles attached to the skin.

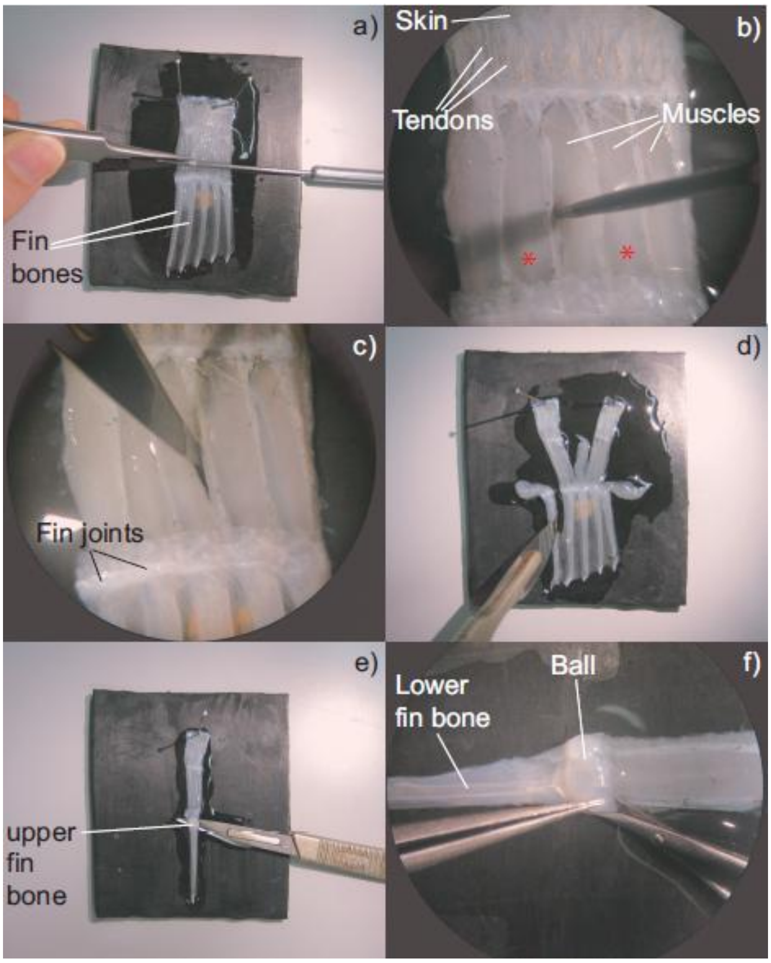

Figure S3. Photographs of: (**a**) upper skin, fin muscles and fin bones of the original muscle block, pinned skin down, illustrating removal of the layer of fin muscles lying next to central layer of bone; (**b**) view down stereoscope, magnification approx. 4.5 times, showing the method for loosening muscles adjacent to those chosen for further dissection (marked *) to allow the skin underneath to be cut; (**c**) cutting of the lower layer of skin around chosen muscles, magnification ~4.5 times; (**d**) separation of muscles by cutting the skin around their edges, between fin joints and down the length of the fin bones; (**e**) separation of the two halves of the fin bone by cutting between them; (**f**) removal of the ball of the fin joint, viewed down the stereoscope at magnification ~4.5 times.

Once the raft of muscles had been cleared of debris, those muscles which would be used for the experiments were chosen, usually two from each raft, making sure there was at least one muscle between them that could be cut into and later discarded. In this example they are marked by red asterisks in Figure S3b. This Figure also shows how the muscles adjacent to those chosen for further dissection were loosened along their edges with a probe. This was to allow the skin below to be cut

on either side of the chosen muscle (see Figure S3c,d). Figure S3e shows how the fin joint and skin between the fin bones was carefully cut to separate the muscles for further dissection. Each fin bone actually comprises two bones which run parallel against each other up to the joint with the main body, where they split to pass on either side of the ball joint. With the muscle pinned so that the upper skin faced down again, the uppermost bone was removed by sliding a scalpel between it and the bone underneath and slicing between them along their length (Figure S3e). The ball of the fin joint was then removed using scissors (Figure S3f).

After this the layer of skin underneath the muscle was removed so that the X-ray and laser beams could pass directly through the muscle. Firstly, at the point where the muscle is attached to the fin bone, a probe was inserted between the muscle and the skin, after carefully loosening the sides of the muscle away from the surrounding tissue (Figure S4a). Scissors were then inserted into the opening, one blade between the muscle and skin and one blade under the skin, to cut the skin close to the fin bone joint (Figure S4b). The connections between the skin and the muscle were cut using scissors (Figure S4c) and the skin removed, close to the tendon attachments at the top of the muscle (Figure S4d). Finally the connections between the fatty tissue layer at the top of the muscle and the muscle were cut (Figure S4e) and the fatty tissue removed (Figure S4f). A small slit was made in the piece of skin at the top of the muscle for the tension transducer hook to pass through. Finally the muscles were pinned out onto individual pieces of rubber (Figure S5) and immersed in a beaker of fresh aerated Ringer solution kept on ice. The muscles could be used without any obvious deterioration of performance for up to 15 h after their dissection.

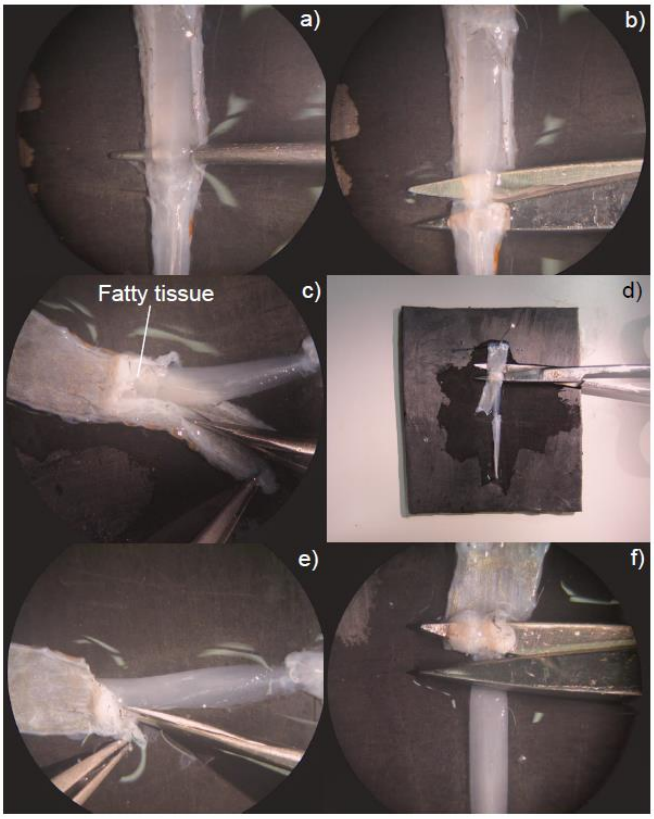

Figure S4. Photographs of: (**a**) a single fin muscle, bone and skin illustrating the loosening of the muscle away from the skin near the fin bone connection; (**b**) method for cutting the skin at the base of muscle near the connection to the fin bone; (**c**) process of cutting connections between the skin and muscle so that skin can be cut at the top of the muscle, close to the tendon attachments, as shown in (**d**); (**e**) process of cutting connections between the muscle and the fatty tissue which covers the top of it so that it can be removed as in (**f**). (**a**–**c**,**e**,**f**) are views down the stereoscope at magnification ~4.5 times that of (**d**).

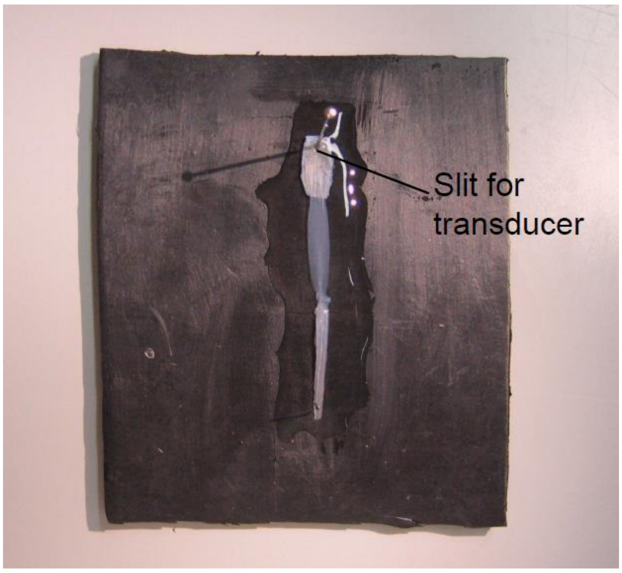

Figure S5. Photograph of a fully dissected Plaice fin muscle preparation. The image shows the slit cut into the skin at the top of the muscle to allow attachment to the tension transducer.

2. Optimising Muscle Contractile Response

Different Ringer solutions were tested prior to the final experiments in order to optimise the contractile response of the Plaice fin muscles. Tetani from muscles in different Ringers (see Table S1) are compared in Figure S6 where it is clear that choice of Ringer can affect the shape of the tetanus including the rise time and relaxation rate. The maximum tension achieved was also affected. The Ringer from James et al. [4] was found to give the best results.

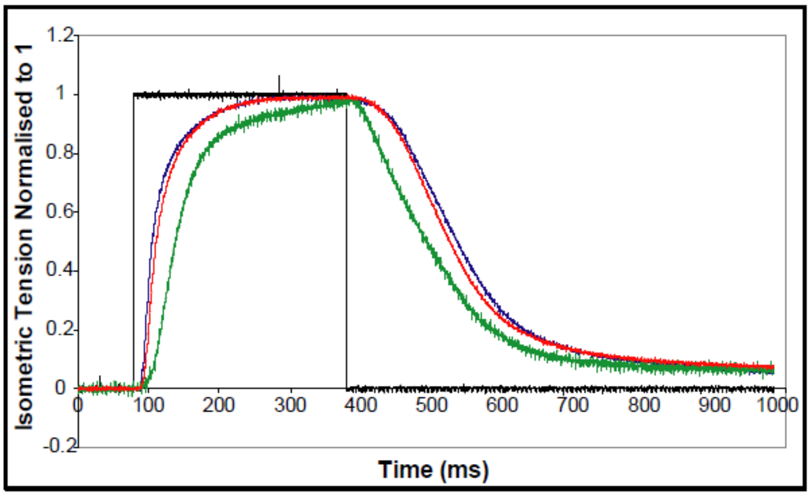

Figure S6. Plot comparing tension time-courses for the first tetanic contractions of three Plaice fin muscles in three different Ringer solutions. The tension time-course from the preparation in the James Ringer is shown in blue, the modified Cobb + Ca in red and the modified Cobb-Ca Ringer in green. (See Table S1 for Ringer compositions). The duration of the stimulation is shown in black.

Table S1. Composition of the 3 different Ringers tested: a Ringer developed for plaice by Cobb et al. [5], a Ringer developed by James et al. [4] for Sculpin and a calcium free Ringer derived from Cobb's recipe by Harford [1].

	Original Cobb Ringer	James Ringer	Modified Cobb—Ca Ringer
**Fish**	**Plaice**	**Sculpin**	**Plaice**
**Chemical**	**Concentration (mM)**	**Concentration (mM)**	**Concentration (mM)**
NaCl	140.7	143	141
KCl	5.2	2.6	5
$MgCl_2$	1.1	1	1
$NaHCO_3$	2.4	6.18	2
NaH_2PO_4	1.8	3.2	2
$CaCl_2$	4.9	2.6	-
Glucose	5	-	5
Na Pyruvate	-	10	-
HEPES Na Salt	-	3.2	-
HEPES	-	0.97	-
pH	8.1	7.4	6.8

3. Mounting the Dissected Muscles

The dissected muscles were mounted in Perspex X-ray chambers similar to the one shown in Figure S7. Each chamber contained a Mylar window in the back of the chamber to allow X-rays to pass into the chamber with the least attenuation. The muscle was positioned across this window. There were two platinum wires, attached to the stimulating pins, which ran on either side of the muscle and parallel to its long axis. These wires transmitted the electrical stimulus to the muscle from the stimulator. On the left side, there was a movable clamp for the fin bone, to hold that end of the muscle preparation secure and stationary. The slot at the top of the chamber allowed the transducer lever arm to enter the chamber (Figure S7b) and the syringe on the right of the chamber allowed air to be bubbled through the Ringer solution contained in the chamber. However, air was not bubbled though the Ringer during experiments in which sarcomere length was being measured because the bubbles caused fluctuations in the laser beams used for the measurements. No deterioration in the response of the muscles was observed when aeration was ceased. The chamber had to be slotted into the muscle control system before the muscle could be mounted. This was so that the transducer lever arm was available for hooking into the slit in the skin. Figure S7b shows the chamber within the control system, with a muscle mounted and the rubber seal and chamber cover screwed in place. The chamber cover also contained a Mylar window to give the smallest attenuation of the X-ray beam as it exits the chamber. Whilst the muscle was being mounted, a small amount of cold Ringer solution was pipetted into the chamber to keep the muscle moist. Once the chamber cover had been screwed in place the chamber was filled with about 10 mL of fresh cold Ringer solution, through the gap at the top where the lever arm enters the chamber. A copper cooling jacket, positioned against the back of the chamber, kept the temperature inside the chamber between 7 and 8 °C for all experiments by circulating a mixture of cold water and anti-freeze continuously through the chamber. The water mixture was cooled and circulated using a Neslab RTE-7 refrigerated bath.

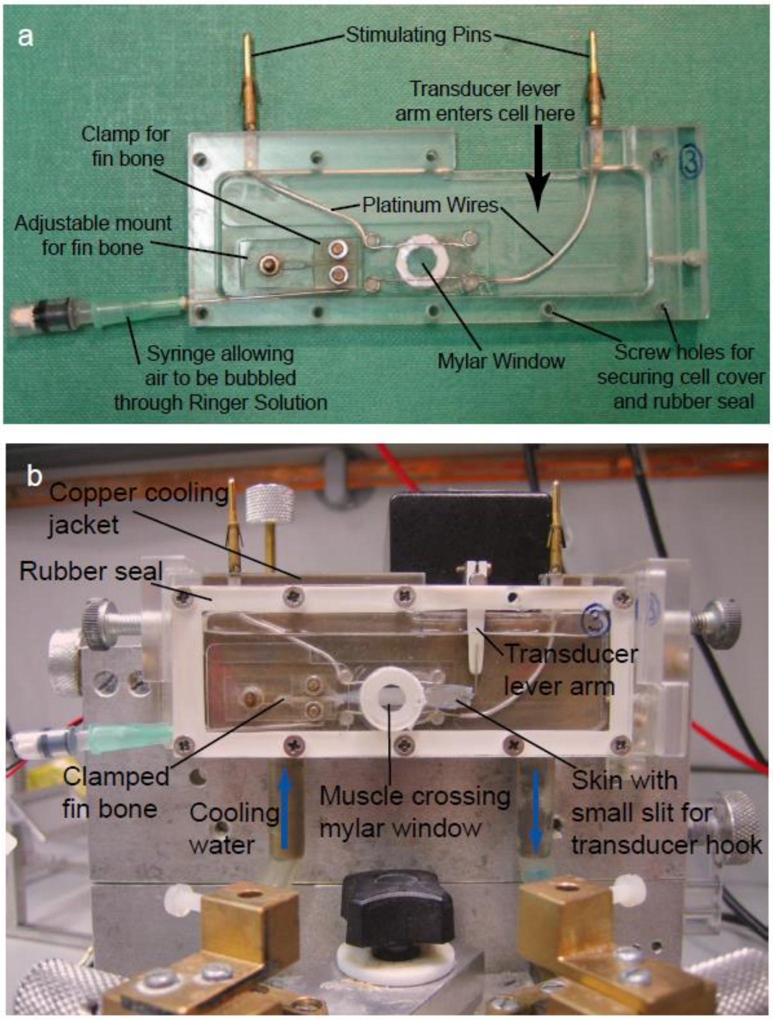

Figure S7. Photograph (**a**) of an X-ray chamber in which plaice fin muscle specimens were mounted. The chamber contains a clamp to hold the fin bone of the specimen in place, a gap in the top to allow the transducer lever arm into the chamber, platinum wires to carry the electrical stimulating signal to the muscle and mylar windows to contain the Ringer solution without attenuating the X-rays as they enter and leave the chamber. Photograph (**b**) of a whole Plaice fin muscle mounted in an X-ray chamber within the muscle control system. The rubber seal and chamber cover were screwed in place and the chamber filled with fresh chilled Ringer solution. A copper cooling jacket was located against the back of the chamber to maintain the temperature of the Ringer at 7 to 8 °C for all experiments. The hooked transducer lever arm entered through the top of the chamber and passed through the small slit in the skin attached to one end of the muscle.

4. Designing and Testing the Sarcomere Length Control System

4.1. Transducer

The transducer used in this system was an Aurora Scientific Inc. (Aurora, ON, Canada) 300B Lever Arm Tension and Length Transducer. A transducer control unit was required to convert the output from the transducer into voltage signals proportional to distance moved by the transducer lever arm and tension exerted on it by the muscle preparation. The unit also allowed the position of and tension on the lever arm to be controlled via external inputs. For all experiments reported here the transducer was set to isometric mode. When sarcomere length control was required, the control signal was fed into the transducer control unit via the length control input. This imposed small deviations of the lever arm from the fixed isometric position to try to compensate for any change in sarcomere length detected by the system. The transducer control unit used in this system was an Aurora Scientific Inc. Dual Mode Lever System.

4.2. Light Source

The light source used for optical diffraction measurements was a Power Technology Inc. (Alexander, AR, USA) PM04 (635-10)LO Diode Laser of wavelength 635 nm and maximum power 5 mW. Both first order laser diffraction peaks from the muscle specimen were monitored by the control system by means of two On-Trak Photonics Inc. (Irvine, CA, USA) 1L20 one-dimensional position sensitive detectors (PSDs) with active areas of 20 mm × 3 mm coupled to two On-Trak Photonics Inc. OT-301SL Single Axis Position Sensing Amplifiers. The circuit box also contained a differential amplifier which subtracted the output of one amplifier from the output of the other. The output signals from the system such as the muscle tension and laser reflection positions were monitored on a digital oscilloscope.

4.3. Stimulator

The stimulator used was D330 Digitimer Ltd. (Welwyn Garden City, UK) Multistim System, which produced trains of negative, positive or alternating square voltage waves and could be externally gated by the computer. The parameters for the stimulus were optimised as above to frequency 140 Hz, pulse width 0.08 ms and amplitude 17 V. The duration of the stimulus was set to 250 ms by a gating signal provided by the data acquisition system. Computer control and data acquisition were also implemented for the system.

4.4. The Laser and Optics

The diode laser used to produce the optical diffraction patterns from the muscle specimens contained an internal line-generating optic that was configured to produce a beam diverging in the vertical direction but focused in the horizontal direction at the position of the PSDs. A 50 mm focal length cylindrical lens was positioned directly in front of the laser to focus the beam in the vertical direction, with the focus lying at the position of the muscle sample. This produced a laser beam with a similar shape and size to the synchrotron beam, ensuring that the part of the muscle for which sarcomere length would be measured and controlled was the same as that being probed by X-rays. Since the muscle control system would only control the sarcomere length in the area of the muscle illuminated by the laser, it was important for the laser and X-ray beams to be co-linear as they passed through the sample. This was achieved by the use of a mirror which reflected the laser beam to bring it along the same path as the X-ray beam, but which transmitted the X-ray beam unaffected. The position of the mirror can be seen in Figure S8.

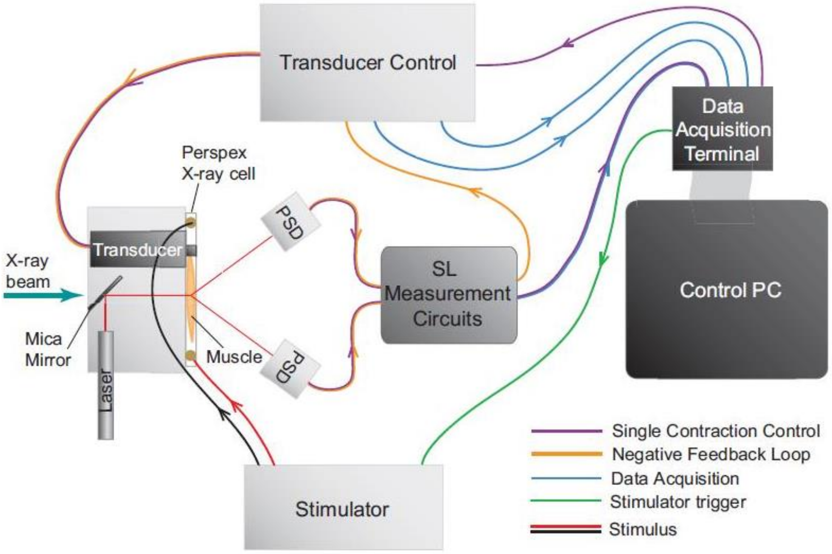

Figure S8. Schematic diagram of the muscle control system, viewed from above. The connections for the two different types of SL control are shown in orange and purple. PSD: Position Sensitive

Detectors. The laser and X-ray beams are shown, following the same path through the muscle specimen.

A prototype test mirror was composed of a sample of polyethylene terephthalate (or mylar) film of 35 μm thickness, coated with an aluminium flash of approximately 0.1 μm thickness. The film was held flat by gluing it onto a metal washer. To look at the X-ray attenuation of the coated polymer film, and any possible X-ray scattering the polymer might cause, the mirror was tested using an in-house X-ray generator of wavelength 1.5418 Å. The experimental setup is shown in Figure S9. A pin wheel rotating at 10 Hz was used to chop the X-ray beam to allow averaging of the recorded X-ray beam intensity, after passing through the sample. The intensity of the X-ray beam was recorded using a PIN diode whose output was viewed and saved on a digital phosphor oscilloscope. The results from these tests gave a transmission factor of 0.988 ± 0.002 for the aluminium coated polymer film mirror. No scattering rings or reflections were visible in the 2D X-ray diffraction patterns at the spatial resolution used for the time-resolved X-ray diffraction experiments in this project. A new film mirror was produced from polyethylene terephthalate film of thickness 2 μm with an aluminium flash of approximately 0.1 μm. The film was glued to a metal washer to hold it taught and to provide a convenient mount and used during our experiments.

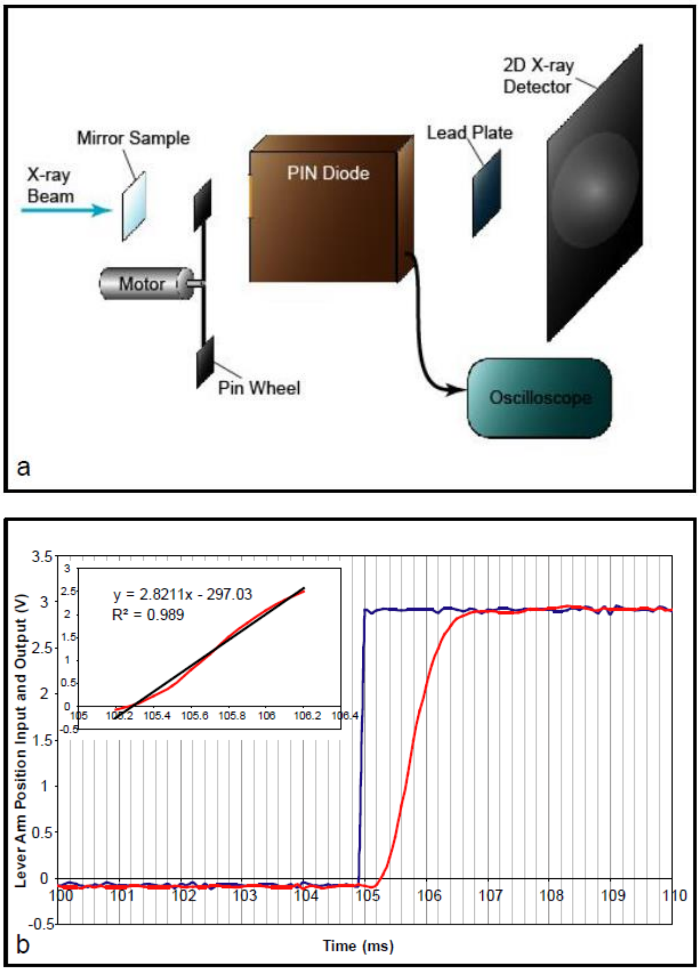

Figure S9. Experimental setup (**a**) for measuring the X-ray transmission factors and possible scattering rings or reflections of the aluminium coated mica and aluminium coated polymer film optical mirrors. A pin wheel connected to a motor, rotating at 10Hz, was placed in the X-ray beam to chop the beam. This allowed an average beam intensity to be calculated for each sample tested. The X-ray beam intensity was measured using a PIN diode, the output signal from which was displayed and recorded on an oscilloscope. A lead plate was placed after the PIN diode in the beam path to prevent the full beam power damaging the 2D X-ray detector. The 2D detector was used to record the diffraction patterns from the two mirrors, with the PIN diode and pin wheel removed. This allowed the mirrors

to be checked for diffraction rings or reflections which might interfere with recording of muscle X-ray patterns at the synchrotron. The wavelength of the X-ray beam was 1.5418 Å; (**b**) Plot to show the response time of the transducer lever arm to an imposed length step. The transducer response is shown in red and the length step is shown in blue. The inset plot shows the straight line fit (black) to the transducer response (red).

4.5. The Detectors

The position sensitive optical detectors were used to measure the changes in position of the two first order laser diffraction reflections from the muscle specimen. Before use they were tested for linearity, intensity independence and response time and calibrated so that sarcomere length changes could be measured as well as controlled.

Using Two Diffraction Orders

The use of both first order laser diffraction peaks for SL measurement required careful arrangement of the two detectors. To produce the required signals each PSD was configured so that a beam positioned at the centre of the active area would give a 0V position output. The negative and positive sides of the detectors were then aligned so that they were in the same position for both PSDs. Since the differential amplifier took the difference of the two PSD output signals, this setup allowed any movements of the two beams in the same direction, as a result of gross pattern movement and not sarcomere length change, to be cancelled out and give a 0V differential amplifier output. Figure S10 shows the arrangement of the detectors. The differential amplifier was configured to subtract the output of PSD 1 from the output signal of PSD 2

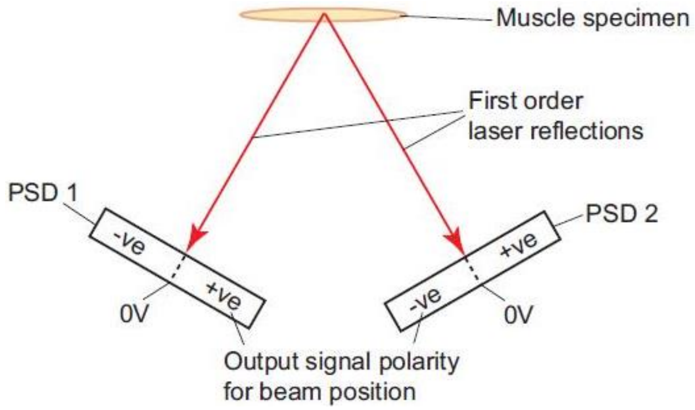

Figure S10. Schematic diagram of the arrangement of the optical detectors within the laser diffraction system, from above, showing their configuration with respect to output polarity for beam position. The detectors were configured so as to give a position output of 0 V for a reflection positioned at the centre of their active area.

Both first order laser diffraction peaks were used for SL measurement rather than just one because measurement of the zero order showed that the diffraction pattern as a whole can move during contraction, see Figure S11. This movement in the whole diffraction pattern would cause an erroneous reading in the SL measurement if only one order was measured.

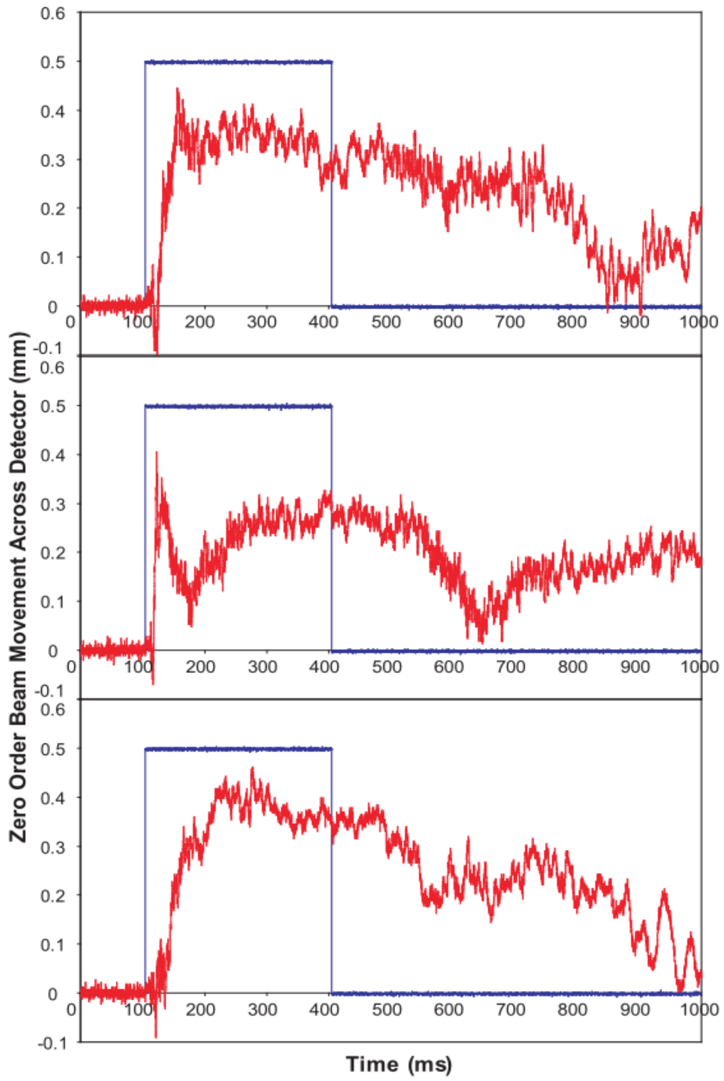

Figure S11. Plots showing the movement of the zero order beam from the optical diffraction pattern in millimetres (red) during an end held isometric contraction in Plaice fin muscle compared to the gating signal of the stimulus causing the muscle to contract (blue). Stimulus duration 300 ms. The three different plots come from three different muscle preparations.

4.6. Tension Transducer

The tension output of the transducer was calibrated by hanging weights off the lever arm and plotting the force exerted against the tension voltage from the transducer controller. However, for some of the larger muscles, the tension voltage signal saturated the data acquisition card. Therefore, a potential divider was built to reduce the signal to 3/4 of its raw value before it was fed into the acquisition card. The tension voltage was then calibrated with the potential divider in place. The calibration plot showed a linear relation between force applied and output voltage.

To test the transducer response to an imposed length step, a rubber band was connected to the lever arm at one end and held stationary at the other. A 3 V length step was then applied to the transducer lever arm position input because this was the size of a typical sarcomere length control signal which would be fed into it. The length output was monitored during this step and the results are shown in the plot in Supporting Figure S9b. The transducer lever arm position could not change as fast as the imposed length step signal, showing a 1.6 ms lag to the 0.1 ms 0 to 3 V length step. The transducer response was fitted by a straight line to find its gradient as shown by the inserted plot in Figure S9b, (inset). It was found that the position response factor of the transducer was therefore 17 times faster than the fastest SL signal which would be applied to it. During synchrotron experiments,

the muscle control system installed on the X-ray beamline was externally controlled using a computer control and acquisition program written within the software Testpoint 3.4.

5. Setting up the Experiments

After mounting the muscle as in Figure S6b the sarcomere length was set using the system in Figure S12 where a mirror was used to project the laser diffraction pattern from the muscle onto a calibrated screen at the top. This provided a longer 'camera' length with which to view the diffraction pattern, hence more accurate setting of the sarcomere length.

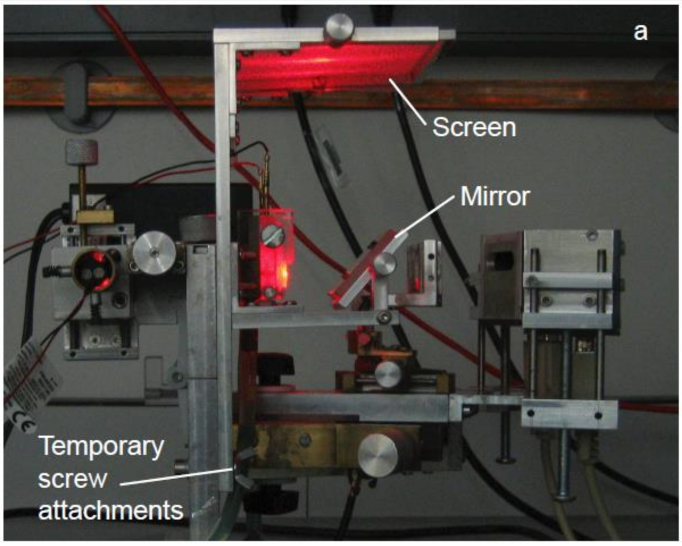

Figure S12. Photograph showing the removable screen and mirror to view the laser diffraction pattern as viewed from the side. The screen allowed sarcomere length to be set accurately at the start of each experiment and is made from graph paper; S6. Performance of the Sarcomere Length Control System.

Figure S13 shows the SL change measured by the system during a tetanic contraction in Plaice fin muscle. The blue traces show SL change without SL control and the red traces show SL change with SL control. A clear reduction in the size of SL change can be seen when the system is acting to limit the change.

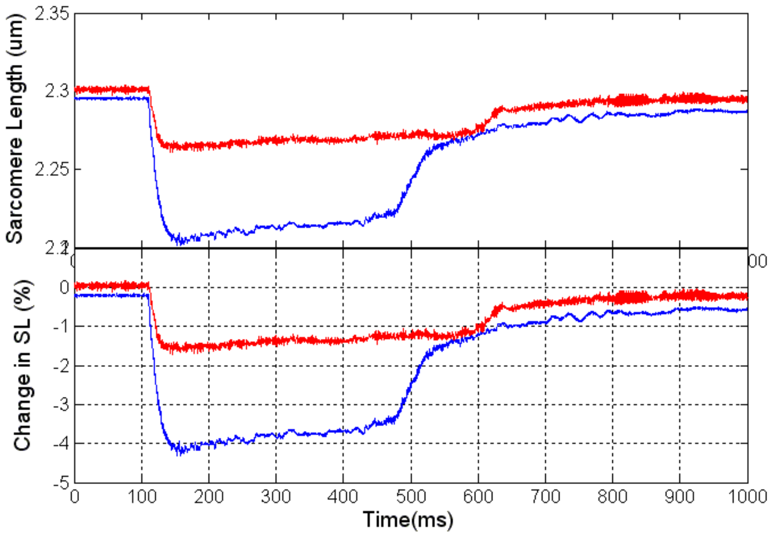

Figure S13. The upper plot shows the absolute sarcomere length change accompanying a tetanic contraction in Plaice fin muscle with SL control (red line) and without SL control (blue line). The lower plot shows the same two traces as above but plotted in terms of percentage SL change.

6. Modelling the Data

For the purposes of the timecourse calculations the relative values of the equatorial intensities for rigor fish muscle were determined from diffraction patterns recorded by the authors at the ESRF [6]. These will be described in detail elsewhere. Figure S14 shows an example of the equatorial profile from one of these patterns. Since the active and rigor patterns were not recorded from the same muscles, the scaling between the patterns is uncertain. We used a scaling factor between rigor and resting diffraction patterns based on the results of Yu and Brenner [7] from skinned rabbit muscle fibres and Squire et al. [8] on skinned fish muscle fibres, where patterns from the same or similar fibres could be recorded from the resting, activated and rigor states. However, this scaling also affects the modelling calculations reported here in that, if the contractile cycle does contain rigor-like bridges and, for example, if the scaling slightly underestimates the strength of the rigor diffraction pattern, then the modelled rigor population would need to be increased to compensate (and vice versa). The modelling would still work, but the estimated populations might be slightly in error. However, we believe that on this basis any population error is not likely to exceed a few %. The relative structure amplitudes used in the modelling are shown in Table S2.

Table S2. Relative structure amplitudes (not Lorentz corrected) used in the modelling (NB Figure 9 shows intensity timecourses).

Reflection	Rest	Active Plateau	Rigor
10	10.00	8.14	6.32
11	5.10	8.54	9.61
20	2.79	2.21	4.81
21	2.13	1.32	2.80
30	1.76	2.12	1.62
22	0	0	0
31	0	2.1	-
40	0	1.64	-
32	0	1.55	-

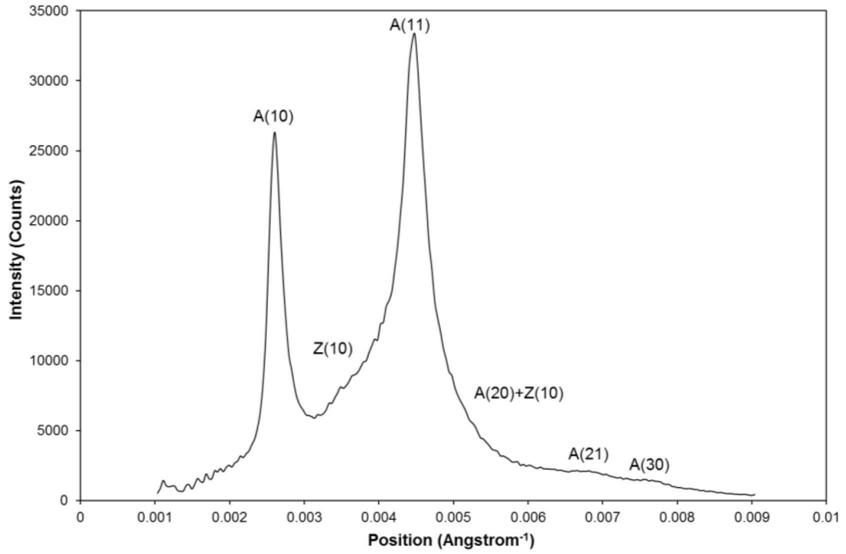

Figure S14. Equatorial profile from rigor plaice muscle recorded by the authors at the ESRF [6]. The pattern has been processed as for the time resolved data, described in the main text.

References

1. Harford, J.J. Diffraction Analysis of Vertebrate Muscle Cross-bridge Arrangements. Ph.D. Thesis, Imperial College, London, UK, 1984.

2. Chayen, N.; Freundlich, A.; Squire, J.M. Comparative histochemistry of a flatfish fin muscle and of other vertebrate muscles used for ultrastructural studies. *J. Muscle Res. Cell Motil.* **1987**, *8*, 358–371.
3. Chayen, N.; Rowlerson, A.; Squire, J.M. Fish muscle structure: Fibre types in flatfish and mullet fin muscles using histochemistry and antimyosin antibody labelling. *J. Muscle Res. Cell Motil.* **1993**, *14*, 533–542.
4. James, R.S.; Cole, N.J.; Davies, M.L.F.; Johnston, I.A. Scaling of intrinsic contractile properties and myofibrillar protein composition of fast muscle in the fish Myoxocephalus scorpius L. *J. Exp. Biol.* **1998**, *201*, 901–912.
5. Cobb, J.L.S.; Fox, N.C.; Santer, R.M. A specific ringer solution for the Plaice. *J. Fish Biol.* **1973**, *5*, 587–591.
6. Eakins, F. Physiology and Crossbridge Mechanism of Bony Fish Muscle. Ph.D. Thesis, Imperial College London, London, UK, 2008.
7. Yu, L.C.; Brenner, B. Structures of actomyosin crossbridges in relaxed and rigor muscle fibres. *Biophys. J.* **1989**, *55*, 441–453.
8. Squire, J.M.; Podolsky, R.J.; Barry, J,S.; Yu, L.C.; Brenner, B. X-ray diffraction testing for weak-binding crossbridges in relaxed bony fish muscle fibres at low ionic strength. *J. Struct. Biol.* **1991**, *107*, 221–226.

© 2016 by the authors. Submitted for possible open access publication under the terms and conditions of the Creative Commons Attribution (CC-BY) license (http://creativecommons.org/licenses/by/4.0/).
